# Corridors or risk? Movement along, and use of, linear features varies predictably among large mammal predator and prey species

**DOI:** 10.1111/1365-2656.13130

**Published:** 2019-12-04

**Authors:** Melanie Dickie, Scott R. McNay, Glenn D. Sutherland, Michael Cody, Tal Avgar

**Affiliations:** ^1^ Alberta Biodiversity Monitoring Institute University of Alberta Edmonton AB Canada; ^2^ Wildlife Infometrics Inc. Mackenzie BC Canada; ^3^ Cenovus Energy Inc. Calgary AB Canada; ^4^ The Department of Wildland Resources Utah State University Logan UT USA

**Keywords:** habitat selection, human disturbance, iSSA, movement, predator‐prey dynamics, road ecology

## Abstract

1. Space‐use behaviour reflects trade‐offs in meeting ecological needs and can have consequences for individual survival and population demographics. The mechanisms underlying space use can be understood by simultaneously evaluating habitat selection and movement patterns, and fine‐resolution locational data are increasing our ability to do so.
2. We use high‐resolution location data and an integrated step‐selection analysis to evaluate caribou, moose, bear, and wolf habitat selection and movement behaviour in response to anthropogenic habitat modification, though caribou data were limited. Space‐use response to anthropogenic linear features (LFs) by predators and prey is hypothesized to increase predator hunting efficiency and is thus believed to be a leading factor in woodland caribou declines in western Canada.
3. We found that all species moved faster while on LFs. Wolves and bears were also attracted towards LFs, whereas prey species avoided them. Predators and prey responded less strongly and consistently to natural features such as streams, rivers and lakeshores. These findings are consistent with the hypothesis that LFs facilitate predator movement and increase hunting efficiency, while prey perceive such features as risky.
4. Understanding the behavioural mechanisms underlying space‐use patterns is important in understanding how future land‐use may impact predator–prey interactions. Explicitly linking behaviour to fitness and demography will be important to fully understand the implications of management strategies.

## Graphical abstract

The authors provide a framework to combine habitat selection and movement behaviour to understand the mechanisms behind space‐use patterns. They then apply this framework to a predator–prey system of high socio‐economic value in Canada, woodland caribou, to understand caribou, moose, bear and wolf space use in relation to human habitat alteration.

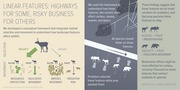

## 1 INTRODUCTION

Animals require a variety of habitats to meet fitness requirements, such as finding food or avoiding predation, resulting in dynamic space use reflecting these trade‐offs (Rosenzweig, 1991). Decomposing habitat functionality is often inferred through habitat selection studies and metrics such as ‘use’, ‘selection’ or ‘avoidance’. Habitat use can have direct consequences to individual fitness and population dynamics, prompting substantial effort into understanding space‐use patterns (Boyce, Vernier, Nielsen, & Schmiegelow, 2002). Individuals may select habitats that provide forage, refuge from predators, or facilitate movement (Avgar, Mosser, Brown, & Fryxell, 2013; Dickie, Serrouya, McNay, & Boutin, 2017). Conversely, individuals may avoid habitats that impede movement or impose predation ‘risk’ (Droghini & Boutin, 2017; Prokopenko, Boyce, & Avgar, 2016). Space‐use patterns thus reflect both movement and habitat functionalities.

Novel landscapes created by anthropogenic habitat modification alter space use of both predator and prey, having implications for species interactions (Fahrig, 2003; Kareiva, 1987). However, responses to novel landscapes are inconsistent and difficult to predict. Anthropogenic habitat modification can increase encounter rates between predators and prey by facilitating predator movement (McKenzie, Merrill, Spiteri, & Lewis, 2012). Alternatively, predators may avoid human‐modified habitats when humans frequent them, leaving spatial refugia for prey species more tolerant of human activity (Berger, 2007; Muhly, Semeniuk, Massolo, Hickman, & Musiani, 2011). Other prey species avoid human‐modified habitats, consistent with a predation risk reduction response (Kauffman et al., 2007). Understanding varying species responses is important for developing effective management actions.

Understanding the mechanisms underlying space use can be achieved with advances in habitat‐use studies which incorporate movement behaviours (Avgar, Potts, Lewis, & Boyce, 2016; Schick et al., 2008). Behavioural responses via use, selection or movement examined independently may be misinterpreted without being put into context with the other patterns. For example, animals moving slowly through certain habitats are consistent with attraction (Knegt, Hengeveld, Langevelde, Boer, & Kirkman, 2007) or movement impediment (Avgar et al., 2013; Fuller, 1991), whereas fast movements are consistent with either an escape from risk or facilitated movement (Dickie, Serrouya, Demars, Cranston, & Boutin, 2017; Frair et al., 2005). By simultaneously evaluating movement and habitat use, coupled with increasing spatio‐temporal resolution in locational data, we can begin to clarify the mechanisms that determine space use and the likelihood of interactions among species (Avgar et al., 2016; Fortin et al., 2005).

**Figure 1:**
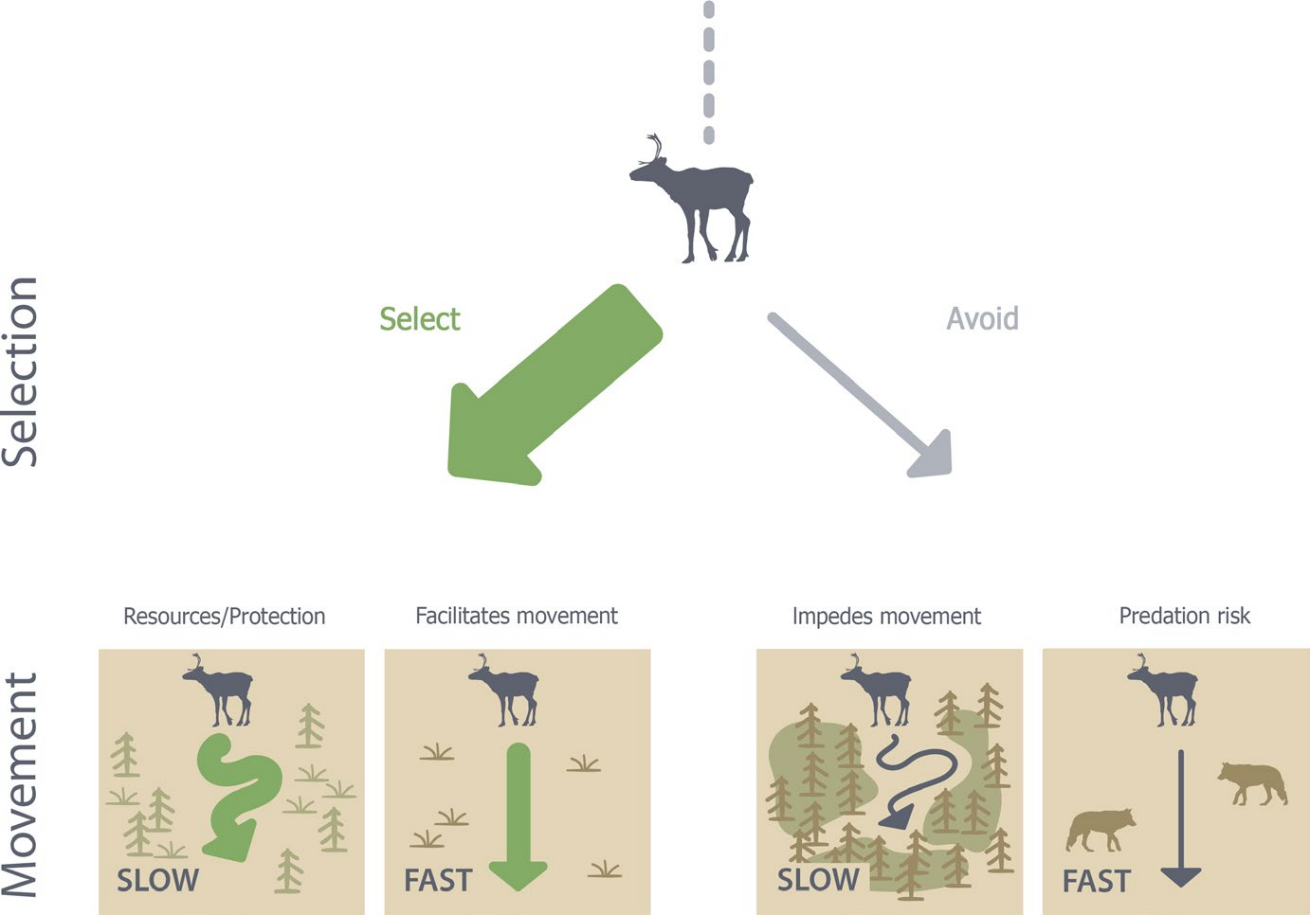
Conceptual figure outlining how incorporating movement into habitat selection study can further clarify animal responses to habitats. For example, habitats selected by an animal may provide resources or protection, leading to slower movements, or can facilitate travel, leading to faster movements. Habitats avoided by an animal may impede travel, leading to slower movements, or may be associated with higher predation risk, leading to faster movements. These patterns can be used to generate predictions about movement behaviours responses if they are hypothesized to provide resources or protection, be used as travel corridors, impede movement or are associated with risk

We provide a framework to combine habitat selection and movement behaviour (Avgar et al., 2016) to understand the mechanisms behind space‐use patterns (Figure 1) and apply it to a predator–prey system of high socio‐economic value in Canada. We postulate that, on average and at a fine spatial scale, habitats that provide resources or protection are likely to be selected by an animal, coupled with slow and tortuous movements. Habitats that facilitate movement are likely to be selected, but movement within such habitats would be fast and direct (reflecting their functionality). Conversely, habitats that pose an impediment to movement are likely to be avoided and coupled with slow tortuous movements. Finally, habitats associated with higher predation risk are likely to be avoided, and movement within such habitats should be fast and direct (to reduce risk exposure). In many systems, the interplay between habitat selection and movement is largely unknown across species, leaving gaps in our understanding of the mechanisms underlying inter‐species responses to human disturbances.

Human disturbance, linked to increased predation, is hypothesized to be a factor leading to woodland caribou *Rangifer tarandus caribou* population declines across North America (Hervieux et al., 2013; Sorensen et al., 2008; Vors, Schaefer, Pond, Rodgers, & Patterson, 2007). Disturbance is linked to woodland caribou declines via an increase in the extent of young seral forest in turn increases the densities of ungulates such as moose *Alces alces* (Osko, Hiltz, Hudson, & Wasel, 2004; Schneider & Wasel, 2000) and therefore the density of wolves *Canis lupus*, which incidentally prey on caribou (the apparent competition hypothesis; Holt, 1977). Additionally, predator use of anthropogenic linear features (LFs) such as roads, pipelines, railroads and seismic lines (long narrow cutlines created for oil and gas exploration) increases predator search rates and facilitates access into caribou habitat, thus increasing the likelihood of incidental caribou kills (DeMars & Boutin, 2017; Dickie, Serrouya, McNay, et al., 2017; Houle, Fortin, Dussault, Courtois, & Ouellet, 2010; James & Stuart‐Smith, 2000). While the influence of human disturbance on behavioural responses by wolves is increasingly well documented, the responses of other key species involved in caribou declines, such as moose, bears and caribou themselves, are less developed (but see Berger, 2007; DeMars & Boutin, 2017; Mumma, Gillingham, Johnson, & Parker, 2017; Serrouya et al., 2017; Tigner, Bayne, & Boutin, 2014; Vistnes & Nellemann, 2008). These knowledge gaps and inconsistencies in responses observed across temporal scales of analysis highlight the need to clarify the mechanisms underlying space‐use patterns using high‐resolution data. Without this understanding, our ability to predict long‐term population responses as a result of land management decisions, including approaches to disturbance and restoration, is challenged.

The objective of this study is to evaluate wolf, black bear, moose and caribou responses to anthropogenic LFs, attempting to determine whether these features are perceived as movement corridors, foraging habitats or as sources of risk. To this aim, we used high‐resolution positional data allowing us to designate movement steps as being on or off LFs. If anthropogenic LFs serve as movement corridors, we predict that individuals select to move towards these features and move faster while on them. In our study system, we expect to observe these patterns for wolves, a wide ranging predator that has been shown to rely on LFs for movement (Dickie, Serrouya, McNay, et al., 2017; McKenzie et al., 2012). If anthropogenic LFs are associated with risk, we predict that individuals avoid these features and move faster while on them. In our system, we expect to observe these patterns in both ungulate species (caribou and moose), with a magnitude reflecting their respective vulnerability to wolf predation (DeMars & Boutin, 2017; Mumma et al., 2017). Lastly, if anthropogenic LFs provide subsidies, as is the expectation for bears, another dominant predator in our system (Dawe, Filicetti, & Nielsen, 2017; Finnegan, MacNearney, & Pigeon, 2018; Tigner et al., 2014), we predict that individuals select these features and move slowly when they are on them. While LFs are predicted to provide subsidies that would also attract moose, we predict LFs are more strongly associated with risk. Additionally, whereas it is possible that LFs also represent risk by human encounters, the majority of anthropogenic LFs within northeastern Alberta receive relatively low human‐use (the majority of these features are unmaintained cutlines such as seismic lines and pipelines, with maintained roads being much rarer; Dickie, Serrouya, McNay, et al., 2017; Dyer, O’Neill, Wasel, & Boutin, 2002; Latham, Latham, McCutchen, & Boutin, 2011), and hunter harvest pressure for these species in this region is low (hunter harvest reports can be found at https://mywildalberta.ca/hunting/hunters-harvest.aspx).

To assess the relative importance of LFs, we also compare the responses to anthropogenic LFs to responses to riparian habitats (rivers, streams and lakeshores). Because wolves are also known to use these features for travel (Latham, Latham, Boyce, & Boutin, 2011; Newton et al., 2017), we predict that wolves select riparian areas and move faster in them. Because riparian areas likely provide an abundance of forage resources (MacCracken, Ballenberghe, & Peek, 1993), we predict moose and bears select riparian areas and move slower in them. Finally, because caribou rely on spatially separating themselves from other ungulates such as moose (James, Boutin, Hebert, & Rippin, 2004), we predict caribou avoid riparian areas and move faster in them.

## 2 MATERIALS AND METHODS

### 2.1 Study area description

**Figure 2:**
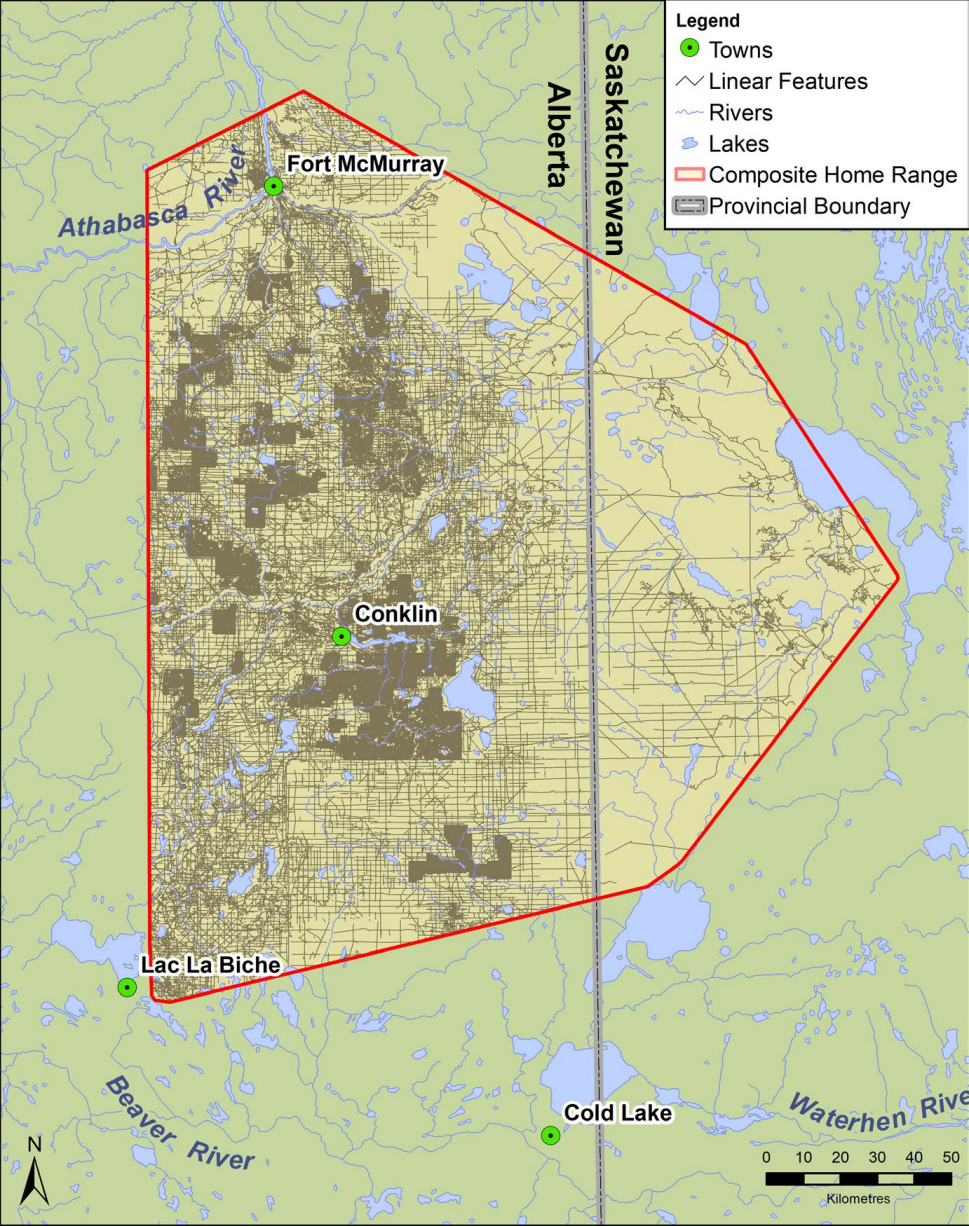
Study area map depicting anthropogenic linear features (LFs) (grey shading), rivers and lakes within the composite 100% minimum convex polygons (red) of collared caribou, moose, black bears and wolves

The 7,826 km^2^ study area straddles the Alberta and Saskatchewan boundary between 55°00′ and 56°00′ N/109°05′ and 111°08′ W (Figure 2). The climate is characteristic of the Boreal Plains Ecozone, with low precipitation (~450 mm/year). The area has limited topographic relief and occurs within the Central Mixedwood Subregion. A mixture of upland forests is interspersed with bog and fen peatland complexes. Ungulate species in the area include moose, woodland caribou and white‐tailed deer *Odocoileus virginianus*. Predators of those ungulates include grey wolves, coyote *Canis latrans*, black bears and lynx *Lynx canadensis* (relative abundances presented in Fisher & Burton, 2018). Other important prey include beaver *Castor canadensis* and snowshoe hare *Lepus americanus*.

### 2.2 Habitat classification

Landcover was classified into polygons using provincial vegetation inventory data (Alberta Vegetation Inventory and Saskatchewan Forest Vegetation Inventory), provincial fire polygon data and wetland classification data in Alberta (Ducks Unlimited Canada, 2011) or Saskatchewan ecosite data (Lane Gelhorn, *pers comm*.). Data gaps were supplemented with Common Attribute Schema for Forest Resource Inventory (CASFRI; Cumming et al., 2010) and Earth Observation For Sustainable Development of Forests (EOSD). Wetlands were identified where vegetation inventory data have soils classified as ‘moist’ or using wetland classifications defined in other datasets. Per cent composition of tree species was used to specify uplands as coniferous (>70% conifers), deciduous (>70% deciduous species) or mixedwood. Landcover was classified into broad categories that we considered biologically relevant: coniferous, deciduous and mixedwood, wetlands or other (including water, unvegetated areas, unclassified burns and unknown landcover categories, see Appendix S1 for additional description of landcover), making up approximately 23%, 29%, 33% and 15% of the study area, respectively.

Disturbances were manually digitized and visually classified at a 1:15,000 scale using 2012 SPOT imagery (2‐m resolution) and Valtus Views (0.5‐m resolution). Data were augmented with disturbance features created post‐2012 using data from industrial sources. All newly collected data were visually verified using aerial or satellite imagery and attributed by two seasonal time steps per year (November‐April and May‐October) to temporally match animal location data. Disturbances that did not include date of construction were assumed to be present at the beginning of the study. Facilities or clearings with a distinct 'footprint' (e.g. camps, laydowns and borrow pits) were supplied by industrial sources. Well pads were collected as point locations and converted to 60 × 90 m polygons.

While quantifying differences among LF categories was important, each individual feature type was rare on the landscape. Therefore, disturbance features were aggregated a priori based upon their structural similarities: CLI = conventional seismic, low‐grade road, or ice road characterized as approximately 8–20 m wide, long and straight, and relatively frequently used by humans; LIS = low‐impact seismic characterized as <8 m wide, sinuous and often dense; PT = pipeline or transmission line characterized as 20–37 m wide and infrequently used by humans; and TRAN = railway or high‐grade road characterized as approximately 30 m wide and frequently used by humans. We also included polygonal disturbances (industrial facilities and clearings, well pads, and cutblocks) as a separate disturbance type to assess differences in habitat selection and movement behaviour associated with these features. We had insufficient data to evaluate differences across each polygonal disturbance type.

### 2.3 Animal captures and GPS data

Global positioning system (GPS) radio collars programmed to collect fine‐resolution data were deployed on 33 wolves, 35 bears, 4 caribou and 18 moose between December 2012 and May 2015. GPS locations were collected every 15 min in the calving (May and June) period for moose and caribou and the calving and early neonate period (May through July) for bears and wolves. Data from two individuals were removed from analyses due to insufficient sample size (collars collected data at the incorrect sampling frequency), resulting in data for 32 wolves (19 males, 12 females, 1 unknown), 34 bears (18 male, 16 female), four female caribou and 18 female moose. Due to the trade‐off between high temporal resolution GPS data and battery constraints, we were unable to collect long‐term location data with high temporal fix rates year‐round. We targeted the snow‐free period because it is when bears are active and woodland caribou neonates and adults are most susceptible to predation (McLoughlin, Dzus, Wynes, & Boutin, 2003). This period is also when anthropogenic LFs are hypothesized to provide the biggest movement benefit to wolves (Finnegan et al., 2018) and deep, uncompacted snow conditions on unmaintained LFs are less favourable during winter months (Droghini & Boutin, 2017). All GPS data were screened for potential errors by excluding 3‐dimensional locations with a dilution of precision (DOP) >10 and 2‐dimensional locations with DOP > 5 (accounting for <0.3% of the total data from individuals monitored with 15‐min data), as well as based descriptors of movement following the methods of Bjørneraas, Van Moorter, Rolandsen, and Herfindal (2010).

### 2.4 Evaluating responses to disturbances and natural habitat

To evaluate how anthropogenic disturbances and riparian areas influence habitat selection and movement of predators and prey, observed 15‐min movement steps (i.e. straight lines connecting successive GPS locations) were compared to random steps; the former typically used to estimate habitat use, and the latter as an estimate of habitat availability (Boyce et al., 2002). Habitat selection and movement are interlinked, with movement rates influencing selection and vice versa (Avgar et al., 2013, 2016; Fortin et al., 2005). Therefore, integrated step‐selection analysis (henceforth, iSSA) was used to compare observed and random steps to evaluate habitat selection while simultaneously accounting for the differing movement behaviours (Avgar et al., 2016; Prokopenko et al., 2016; Scrafford, Avgar, Heeres, & Boyce, 2018; Viana et al., 2018).

Ten random steps for each observed step were generated using analytical distributions that were parameterized based on the observed animal movement patterns. Observed movement patterns were described using gamma distribution of step length and a von Mises distribution of turning angles, parameterized independently for each species. Parameterization (using package 'circular'; Agostinelli & Lund, 2017) was done based on 'moving' steps only. Moving steps were defined as >20.53 m for wolves and >25.86 m for bears (based on a broken‐stick analysis following the methods of Dickie, Serrouya, Demars, et al., 2017), and >16 m (collar error; Dickie, 2015) for moose and caribou because the result of their broken‐stick analysis revealed a breakpoint occurring below collar error.

Habitat selection was evaluated as a function of landcover and distances to: anthropogenic LFs, polygonal disturbances and riparian features. Habitat attributes at the end of each observed step were compared to attributes at the end of random steps. Distances were transformed using the natural logarithm because we expected animals to respond more strongly to features when they were closer to them, with the response decaying at an unknown rate. Landcover type was included to incorporate differences in animal movement behaviour associated with natural habitat (see Appendix S1 for landcover results and discussion).

Movement was evaluated as functions of habitat attributes along each step, that is if the step was on undisturbed forest (the reference category) compared to anthropogenic LFs, polygonal disturbances and riparian habitat. Observed and random steps were classified as being on anthropogenic LFs or polygonal disturbances if both the start and end points were closer than, or equal to, the buffered width of each feature (transmission lines‐37m; high‐grade roads and railways‐30m; pipelines‐20m; low‐grade roads and ice roads‐12m; conventional seismic‐10m; and low‐impact seismic‐7m) following the methods of Dickie, Serrouya, Demars, et al. (2017). If a step was contained within the buffer of multiple anthropogenic LF classes, that step was assigned to the anthropogenic LF class with the largest width. For example, a low‐impact seismic line along or crossing a transmission line is indistinguishable from the transmission line. Steps that were not classified as being on disturbances were then classified as on or off riparian areas if both their start and end points occurred within 100 m of a river, stream or lakeshore. A buffer width of 100 m reflected topographic banks and animal movement along these features (see Appendix S2 for sensitivity analysis). While we could have classified steps using only information from the start point, as done in other uses of this model structure (Avgar et al., 2016), our approach takes advantage of the high temporal resolution of our data allowing us to be more certain of movement along the habitats of interest.

### 2.5 Statistical modelling

Each individual was modelled separately using conditional logistic regression ('survival' package in r; Therneau 2014). The relative probability of selection was modelled as a function of landcover, distance to anthropogenic LFs, polygonal disturbances and riparian areas. The natural logarithm of step length, cosine of turning angle and their interaction were included as modifiers of the observed movement parameters (used to generate random steps; Avgar et al., 2016). To allow the selection‐free displacement rate to vary with feature type, the interaction between the natural logarithm‐transformed step length and each disturbance type of interest and riparian habitat was also included. For each individual, only disturbance categories that composed >1% of the random points (i.e. availability) were included. No individual had >1% of their random steps on high‐grade roads and railways, so they were removed from all models. The landcover reference category was set to 'other' to evaluate the differences between each of the landcover categories of primary interest.

Individual selection and movement responses were summarized to evaluate consistency in responses based on model coefficients and their 95% confidence intervals (CIs). Selection is defined as occurring if use was higher than availability, resulting in positive selection coefficients, and avoidance if use was less than availability, resulting in negative selection coefficients. CIs overlapping with zero were interpreted as indifference, and non‐overlapping CIs as significant selection/avoidance or an effect on speed, depending on the variable of interest.

We used inverse variance‐weighted linear modelling to obtain population‐level averages for each species (Murtaugh, 2007). The iSSA coefficient values for each variable were used as the response variable in a linear regression, with the variable's availability (to account for potential functional responses; Mysterud & Ims, 1998), and the values of other iSSA coefficients that might be correlated with it as predictors, and the inverse of the estimated variance of the coefficient value as weights (using base r; R Core Team 2014). The resulting intercept from each model can be interpreted as the average response to the feature, accounting for its availability and the uncertainty in each individual's response.

### 2.6 Calculating effect sizes

To understand how strongly habitat features influenced selection, the relative selection strength was calculated (Avgar, Lele, Keim, & Boyce, 2017; Appendix S3). For landcover, we calculated the relative probability of selecting a step ending in one landcover type over another. For disturbance or riparian features, we calculated the relative probability of selecting a step moving towards vs. away from a given feature. To understand how strongly habitat features influenced animal movement rates irrespective of habitat selection, the expected (selection‐free) displacement rates were calculated for each individual by using the iSSA coefficients to adjust the initially observed von Mises and gamma distributions (Appendix S3).

Because species may exhibit sex‐dependent behaviour (Ofstad et al., 2016), we evaluated if the selection for, and movement on, each of our habitat attributes of interest depended on if the animal was male or female (Appendix S4). We also tested for differences during day and night, as a proxy for high and low human‐use, because animals may perceive human activity on disturbances as risky (Appendix S5; Muhly et al., 2011; Theuerkauf, Jedrzejewski, Schmidt, & Gula, 2003; Zimmermann, Nelson, Wabakken, Sand, & Liberg, 2014). We found no evidence that selection for disturbance or riparian areas differed between male and females, nor between day and night.

## 3 RESULTS

Of the used and available steps within our analyses, undisturbed habitat was the most common habitat type (Appendix S6). For all species except caribou, the most common linear feature type was either conventional seismic, low‐impact roads and ice roads or pipelines and transmission lines. All species exhibited the slowest average step length within undisturbed habitat and fastest on anthropogenic LFs. See Appendix S6 for a description of use and availability across steps, availability within each species’ composite home ranges, and average step lengths within each disturbance type. See Appendix S1 for detailed results not pertaining to disturbances or riparian habitat.

### 3.1 Selection for disturbances and riparian areas

**Figure 3:**
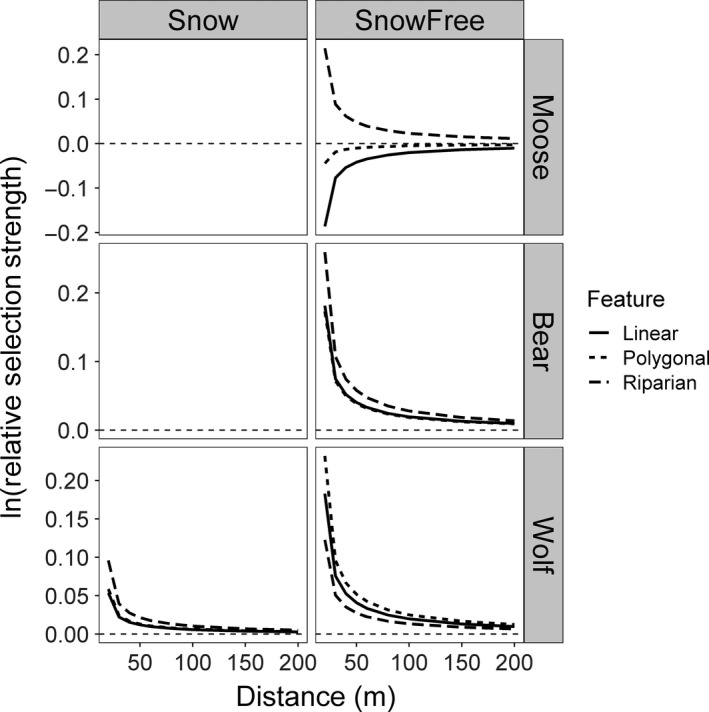
Relative selection strength of anthropogenic linear features (solid line), polygonal disturbances (dotted line) and riparian features (dashed line) by moose, bears and wolves. Dotted horizontal line represents no response, whereas above that line the population is selecting to be closer to that feature than expected, and below the dotted line the population is selecting to be farther from that feature than expected. Caribou are not shown due to insufficient individuals to obtain population‐level averages. The relative selection strength for anthropogenic disturbances and riparian areas was calculated as the expected tendency of moving towards a given feature type compared to away from it (Appendix S3; Avgar et al., 2017)

On average, moose selected to be closer to riparian areas, avoided being closer to anthropogenic LFs and were indifferent to polygonal disturbances (Table 1). For example, when moose were 20 m away from either riparian areas or anthropogenic LFs, they were 1.24 times more likely to move towards riparian areas than away from them, but 1.17 times more likely to move away from anthropogenic LFs than towards them (Figure 3). Many individuals’ CIs overlapped zero, though none of the 18 individuals selected to be closer to anthropogenic LFs (Appendix S7). No individual caribou selected to be closer to polygonal disturbances, anthropogenic LFs and riparian areas. Instead, individual caribou tended to be indifferent to these features (Appendix S7).

**Table 1:** Average prey selection and movement responses to human disturbances and natural habitat. Each individual was modelled separately and then averaged using inverse variance models

Species	Component	Variable	Coefficient	CI	*n*
Moose	Selection	Conifer	−0.116	0.173	18
		Deciduous/Mixedwood	−0.203	0.196	17
		Wetland	0.177	0.176	18
		ln(Distance to LF)	0.050	0.042	18
		ln(Distance to Poly)	0.012	0.088	18
		ln(Distance to RIP)	−0.058	0.045	18
	Movement	ln(SL)	−0.851	0.039	18
		ln(SL):Cos(Turn angle)	0.220	0.017	18
		Cos(Turn angle)	−0.821	0.135	18
		CLI:ln(SL)	0.344	0.104	3
		LIS:ln(SL)	0.060	0.217	2
		PT:ln(SL)	–	–	–
		Poly:ln(SL)	0.113	0.032	2
		RIP:ln(SL)	0.080	0.032	18

Note

The reference categories were ‘other’ landcover and ‘undisturbed forest’ for selection and movement, respectively.

Abbreviations: CLI, conventional seismic, low‐grade roads and ice roads; LIS, low‐impact seismic; PT, pipelines and transmission lines; Poly, polygonal disturbances; RIP, riparian habitat; SL, step length.

Bears selected to be closer to anthropogenic LFs and riparian areas but were indifferent to polygonal disturbances (Table 2). When 20 m away, bears were 1.20 and 1.30 times more likely to move towards anthropogenic LFs and riparian areas, respectively (Figure 3). Wolves were 1.20 and 1.26 times more likely to move towards anthropogenic LFs and polygonal disturbances when 20 m away but were indifferent to riparian areas (Figure 3). While many individuals' CIs overlapped zero, there were few individuals showing significant avoidance for any of the feature types (Appendix S7).

**Table 2:** Average predator selection and movement responses to human disturbances and natural habitat. Each individual was modelled separately and then averaged using inverse variance models by species for each parameter of interest

Species	Component	Variable	Coefficient	CI	*n*
Bear	Selection	Conifer	−0.099	0.131	34
		Deciduous/Mixedwood	0.239	0.141	34
		Wetland	−0.134	0.124	34
		ln(Distance to LF)	−0.049	0.021	34
		ln(Distance to Poly)	−0.047	0.060	34
		ln(Distance to RIP)	−0.070	0.034	34
	Movement	ln(SL)	−1.012	0.037	34
		ln(SL):Cos(Turn angle)	0.676	0.016	34
		Cos(Turn angle)	−3.056	0.129	34
		CLI:ln(SL)	0.279	0.053	13
		LIS:ln(SL)	0.010	0.233	2
		PT:ln(SL)	0.304	0.066	15
		Poly:ln(SL)	0.077	0.045	17
		RIP:ln(SL)	0.015	0.025	34
Wolf	Selection	Conifer	0.281	0.135	32
		Deciduous/Mixedwood	0.268	0.150	32
		Wetland	0.150	0.130	32
		ln(Distance to LF)	−0.049	0.019	32
		ln(Distance to Poly)	−0.063	0.047	32
		ln(Distance to RIP)	−0.033	0.037	32
	Movement	ln(SL)	−0.798	0.037	32
		ln(SL):Cos(Turn angle)	0.551	0.017	32
		Cos(Turn angle)	−2.533	0.134	32
		CLI:ln(SL)	0.301	0.048	21
		LIS:ln(SL)	0.100	0.201	3
		PT:ln(SL)	0.293	0.073	14
		Poly:ln(SL)	0.005	0.039	22
		RIP:ln(SL)	0.112	0.027	32

Note

The reference categories were 'other' landcover and ‘undisturbed forest’ for selection and movement, respectively.

Abbreviations: CLI, conventional seismic, low‐grade roads and ice roads; LIS, low‐impact seismic; PT, pipelines and transmission lines; Poly, polygonal disturbances; RIP, riparian habitat; SL, step length.

### 3.2 Movement on disturbances and riparian areas

**Figure 4:**
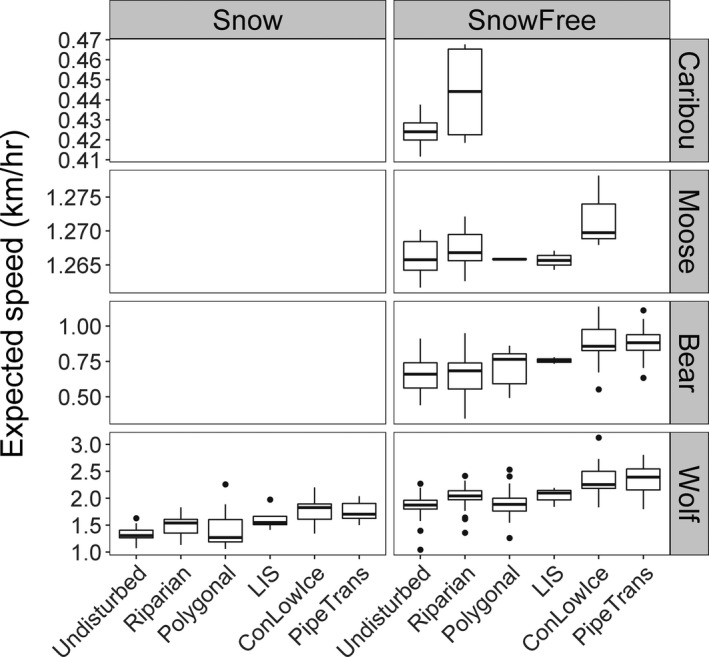
Calculated expected displacement rates (expected speed; km/hr) in undisturbed habitat, riparian features and each disturbance category for caribou, moose, bears and wolves. LIS = low‐impact seismic; ConLowIce = conventional seismic, low‐grade roads and ice roads; PT = pipelines and transmission lines

Moose on average moved faster on conventional seismic/low grade and ice roads, polygonal disturbances and riparian areas than undisturbed forest (Table 1). Almost half (8 of 18) moved faster on riparian, whereas all three moose with sufficient data to estimate the effect of conventional seismic/low grade and ice roads moved faster on them (Figure 4; Appendix S7). Differences in average expected (selection‐free) displacement rates between undisturbed forest and any of the habitats of interest were minimal (Table 3). Individual caribou did not have different displacement rates on riparian areas compared to undisturbed forest (Appendix S7).

**Table 3:** Mean (and standard error; *SE*) calculated expected displacement rates, calculated using the observed von Mises kappa and gamma rate and shape, modified from model coefficients for each individual

Species	Feature	Mean	*SE*
Caribou	Undisturbed	106.09	1.33
	CLI	–	–
	LIS	–	–
	PT	–	–
	Poly	–	–
	RIP	110.92	3.25
Moose	Undisturbed	316.49	0.15
	CLI	317.99	0.32
	LIS	316.42	0.12
	PT	–	–
	Poly	316.46	0.01
	RIP	316.83	0.17
Bear	Undisturbed	164.90	5.21
	CLI	217.35	7.10
	LIS	189.10	1.39
	PT	219.69	5.32
	Poly	178.05	5.22
	RIP	166.53	6.14
Wolf	Undisturbed	462.37	9.99
	CLI	582.45	13.32
	LIS	510.81	8.06
	PT	583.31	12.37
	Poly	476.15	12.59
	RIP	504.50	9.44

Abbreviations: CLI, conventional seismic, low‐grade roads and ice roads; LIS, low‐impact seismic; PT, pipelines and transmission lines; Poly, polygonal disturbances; RIP, riparian habitat.

On average, bears moved faster on conventional seismic/low grade and ice roads, pipelines and transmission lines, and in polygonal disturbances compared to undisturbed forest and riparian areas (Table 2; Figure 4). The majority of bears were consistent with this trend, moving faster on conventional seismic/low grade and ice roads and pipelines and transmission lines (Appendix S7). No bears moved slower on any class of disturbance than in undisturbed forest habitats. The average expected (selection‐free) displacement rates were 52.45 m/15 min, 54.79 m/15 min and 13.15 m/15 min faster on conventional seismic/low grade and ice roads, pipelines and transmission lines and in polygonal disturbances, respectively, compared to movement in undisturbed forest.

Wolves moved significantly faster on conventional seismic/low grade and ice roads, pipelines and transmission lines and riparian areas compared to movement speeds in undisturbed forest (Table 2; Figure 4). Nearly all wolves exposed to these features moved faster on them (Appendix S7). For example, average expected (selection‐free) displacement rates were over 100 m/15 min faster on conventional seismic/low grade and ice roads as well as pipelines and transmission lines than in undisturbed forest (Table 3). Wolf travel speed was not significantly influenced by polygonal disturbances.

## 4 DISCUSSION

### 4.1 Understanding of mechanisms underlying space‐use patterns

We simultaneously evaluated selection and movement responses of multiple predators and prey species to anthropogenic LFs and natural habitats using high‐resolution location data. Wolves and bears selected anthropogenic LFs and moved faster on them, consistent with the hypothesis that predators preferentially use these features to facilitate movement. Counter to expectations, bears did not slow their movements on anthropogenic LFs, potentially reflecting opportunistic predation (Bastille‐Rousseau, Fortin, Dussault, Courtois, & Ouellet, 2011). Moose avoided anthropogenic LFs and moved faster while on them, supporting that moose perceive these features as risk. Also, no individual caribou selected to be closer to anthropogenic LFs, partially supporting the risk‐aversion hypothesis, though with limited data.

Incorporating selection when interpreting movement behaviour was important for understanding mechanisms behind space‐use patterns. For example, wolves, bears and moose moved faster on anthropogenic LFs, but selection of these features by predators was consistent with movement facilitation (Dickie, Serrouya, McNay, et al., 2017), whereas avoidance by prey is consistent with perception of risk (Prokopenko et al., 2016). Likewise, incorporating movement behaviour when evaluating habitat selection was integral in our interpretations of selection patterns. Contrary to our prediction, moose appeared to select riparian areas to facilitate movement instead of selecting them for foraging opportunities. In the absence of incorporating movement behaviour, neutral selection responses of wolves to riparian areas would have under‐represented the importance of these features as movement corridors in our system. Future studies that aim to understand space‐use patterns can incorporate habitat selection and movement responses simultaneously to clarify the mechanisms underlying observed patterns.

It is also important to consider the indirect effects of LF avoidance by prey species. In combination with their unique geometry (narrow but long), LFs may act as movement barriers, effectively fragmenting the landscape (D'Amico, Periquet, Roman, & Revilla, 2016). Moreover, as their avoidance effect extends into the surrounding landscape (Figure 3), their associated habitat loss may be substantially more extensive than their physical footprint (see also Prokopenko et al., 2016). Hence, in a landscape as impacted by LFs as the one studied here (Figure 2), prey species may suffer from reduced access to vital resources as well limited natal dispersal, both with potential consequences for long‐term population health. This effect may be compounded in areas with extensive human activity or high hunting pressure, where disturbances not only increase the risk of predation, but also human access (Fahrig & Rytwinski, 2009). Furthermore, while we found no evidence for sex‐specific responses, differences across females with various reproductive statuses may exist, such that some individuals may be more vulnerable to indirect effects and are an interesting avenue of future research.

Anthropogenic LFs are most similar to streams, rivers and lakeshores; natural features known to facilitate predator movement (Newton et al., 2017). Wolves moved faster while on riparian areas, but did not select these areas, supporting that riparian areas are used as travel corridors, but with less of a movement benefit than anthropogenic LFs. This is consistent with the observation that wolves shift from selecting roads and railways on human‐dominated landscapes to selecting lakeshore and rivers on landscapes where man‐made movement corridors are scarce (Newton et al., 2017). Bears selected riparian areas but did not travel differently while on them. We are therefore unable to distinguish between foraging behaviours (Lyons, Gaines, & Servheen, 2003), movement facilitation or a combination of both. Moose also selected riparian areas, but counter to our predictions, they moved faster on these features, suggesting use of these features as travel corridors. However, the average expected displacement rate on riparian features was only 0.34 km/hr faster than undisturbed habitat and may not be biologically meaningful. Finally, two of four caribou selected riparian areas, but speed was not influenced by this habitat type for any individual. Although inference from this small sample is problematic, these findings are consistent with the notion that caribou may perceive them as less risky than anthropogenic LFs.

We did not find strong selection or movement responses to polygonal features from any of the species of interest. This may reflect differences in responses to the disturbance types included in this broad category. For example, herbivores or omnivores may select young cutblocks and wellpads with young seral vegetation (Rempel, Elkie, Rodgers, & Gluck, 1997), but avoid polygonal disturbances with frequent human activity such as facilities. The limited number of individuals with sufficient steps in polygonal disturbances to estimate selection or movement effects limited our ability to evaluate responses to these features. However, these features were available within the animals' home ranges, suggesting animals may alter their habitat use at broader scales than studied here (see below).

### 4.2 Consequences for predator–prey interactions

If anthropogenic LFs are effective travel corridors for predators, they may increase encounter rates between predators and prey by increasing area searched per unit time (Fryxell, Mosser, Sinclair, & Packer, 2007; Holling, 1959). However, moose, and to a lesser extent caribou, response to these features supports that prey species may be able to spatially separate from preferred predator habitat to reduce risk within certain spatio‐temporal limits (Fortin et al., 2005; Muhly et al., 2011). Given the divergence in behavioural responses to anthropogenic LFs between the predominant predator and prey species in this system, previous studies may have over‐estimated their influence on demography, as suggested by McKenzie et al. (2012). However, encounter rates may still increase if predators are using LFs to travel from patch to patch, or given prey are likely unable to completely avoid these features (DeMars & Boutin, 2017), leaving cues to predators. Conversely, riparian areas were used by both predators and prey, and here may be higher chances for encounters between the species on these features.

Predator–prey theory predicts that increased displacement rate should increase prey encounter and consumption rate (Avgar, Kuefler, & Fryxell, 2011; Holling, 1959). This theory is based on a mean‐field approximation (ideal‐gas model) where the system is well mixed and homogeneous (Avgar et al., 2011; Hutchinson & Waser, 2007). The reality of ecological landscapes rarely meets this assumption, and the distribution of prey and its vulnerability to predation is heterogeneous and dynamic, driven by both habitat heterogeneity (e.g. impacting the prey's resources) and local depletions. Under heterogeneous conditions, the advantages of LFs for wolf hunting extend beyond increased local search rates. For example, LFs may allow wolves (and bears) to travel efficiently, along straight lines, between sparse resource patches, while avoiding lingering in areas they have recently exploited. Recent advances in technology such as the addition of cameras on animal collars (Brockman, Collins, Welker, Spalinger, & Dale, 2017) will clarify these mechanisms and their relative importance for increased hunting efficiency and their direct implications for kill rates. Perhaps more importantly, these analyses should include demographic data such as encounter rates and survival to more fully understand the long‐term population‐level implications of the fine‐scale behavioural responses we studied.

### 4.3 Limitations

Defining the appropriate scales of analysis is imperative in habitat selection studies (Boyce, 2006; Johnson, 1980). Habitat constrains species occupancy, but nested within that constraint, home‐range placement or behaviour within that home range can be influenced by habitat, reflecting a hierarchy in selection patterns (Johnson, 1980). Data used in this study reflect fine‐scale habitat selection and movement. However, the density of LFs may impact habitat selection and movement responses (Johnson, 1980; Mysterud & Ims, 1998; Newton et al., 2017). While moose and caribou home ranges were constrained to the low LF‐density areas in which captures occurred, bears and wolves had larger home ranges that incorporated a variety of disturbance densities (Appendix S6). Given that some animals had disturbances such as high‐grade transportation corridors within their home ranges, but not within their step availability, we suggest that animals are able to modify their movement behaviour at scales larger than that studied here. Future work should evaluate responses across linear feature densities, and analytical scales, to capture the full range of environmental heterogeneity on space use.

### 4.4 Management implications

Our results have implications for directing, prioritizing and predicting the effectiveness of habitat restoration to mitigate the effects of LFs on predator–prey interactions. If LFs are acting as travel corridors from patch to patch, as suggested by our results, restoration activities could focus on areas that connect wetlands (selected by prey species) to upland patches (selected by predator species; Appendix S1), and techniques which reduce movement efficiency. However, both predator and prey may have differing behaviours depending on season, and as such effective management actions should consider the behaviour of all species year‐round. Our results also highlight the importance of understanding bear responses to human‐modified habitats, and how responses influences predation rates.

## AUTHORS' CONTRIBUTIONS

M.D. conceived the ideas, implemented analysis and led paper writing; G.D.S. and M.D. spatially classified data; T.A. supported analysis and interpretation; and M.C. and S.R.M. supported study design and data collection. All authors were critically involved in writing the manuscript.

## Data Availability

Data are available from the Dryad Digital Repository at: https://doi.org/10.5061/dryad.pnvx0k6h8 (Dickie, McNay, Sutherland, Cody, & Avgar, 2019).

## References

[jane13130-bib-0001] Agostinelli, C. , & Lund, U. (2017). R Package “circular”: Circular Statistics (version 0.4 ‐ 93). Retrieved from https://r-forge.r-project.org/projects/circular/

[jane13130-bib-0002] Avgar, T. , Kuefler, D. , & Fryxell, J. M. (2011). Linking rates of diffusion and consumption in relation to resources. The American Naturalist, 178(2), 182–190. 10.1086/660825 21750382

[jane13130-bib-0003] Avgar, T. , Lele, S. R. , Keim, J. L. , & Boyce, M. S. (2017). Relative selection strength: Quantifying effect size in habitat‐ and step‐selection inference. Ecology and Evolution, 7(14), 5322–5330. 10.1002/ece3.3122 28770070 PMC5528224

[jane13130-bib-0004] Avgar, T. , Mosser, A. , Brown, G. S. , & Fryxell, J. M. (2013). Environmental and individual drivers of animal movement patterns across a wide geographical gradient. Journal of Animal Ecology, 82(1), 96–106. 10.1111/j.1365-2656.2012.02035.x 23020517

[jane13130-bib-0005] Avgar, T. , Potts, J. R. , Lewis, M. , & Boyce, M. (2016). Integrated step selection analysis: Bridging the gap between resource selection and animal movement. Methods in Ecology and Evolution, 7(5), 619–630. 10.1111/2041-210X.12528

[jane13130-bib-0006] Bastille‐Rousseau, G. , Fortin, D. , Dussault, C. , Courtois, R. , & Ouellet, J. P. (2011). Foraging strategies by omnivores: Are black bears actively searching for ungulate neonates or are they simply opportunistic predators? Ecography, 34(4), 588–596. 10.1111/j.1600-0587.2010.06517.x

[jane13130-bib-0007] Berger, J. (2007). Fear, human shields and the redistribution of prey and predators in protected areas. Biology Letters, 3, 620–623. 10.1098/rsbl.2007.0415 17925272 PMC2391231

[jane13130-bib-0008] Bjørneraas, K. , Van Moorter, B. , Rolandsen, C. M. , & Herfindal, I. (2010). Screening global positioning system location data for errors using animal movement characteristics. Journal of Wildlife Management, 74(6), 1361–1366. 10.1111/j.1937-2817.2010.tb01258.x

[jane13130-bib-0009] Boyce, M. S. (2006). Scale for resource selection functions. Diversity and Distributions, 12, 269–276. 10.1111/j.1366-9516.2006.00243.x

[jane13130-bib-0010] Boyce, M. S. , Vernier, P. R. , Nielsen, S. E. , & Schmiegelow, F. K. (2002). Evaluating resource selection functions. Ecological Modelling, 157, 281–300. 10.1016/S0304-3800(02)00200-4

[jane13130-bib-0011] Brockman, C. J. , Collins, W. B. , Welker, J. M. , Spalinger, D. E. , & Dale, B. W. (2017). Determining kill rates of ungulate calves by brown bears using neck‐mounted cameras. Wildlife Society Bulletin, 41(1), 88–97. 10.1002/wsb.733

[jane13130-bib-0012] R Core Team (2014). R: A language and environment for statistical computing. Vienna, Austria: R Foundation for Statistical Computing.

[jane13130-bib-0013] Cumming, S. , Schmiegelow, F. , Bayne, E. , & Song, S. (2010). Canada’s forest resource inventories: Compiling a tool for boreal ecosystems analysis and modelling. Version 1.0. Technical Report. Retrieved from http://www.borealbirds.ca/files/technical_reports/CAS_Backgrounder_v1.0.pdf

[jane13130-bib-0014] D'Amico, M. , Periquet, S. , Roman, J. , & Revilla, E. (2016). Road avoidance responses determine the impact of heterogeneous road networks at a regional scale. Journal of Applied Ecology, 53, 181–190. 10.1111/1365-2664.12572

[jane13130-bib-0015] Dawe, C. A. , Filicetti, A. T. , & Nielsen, S. E. (2017). Effects of linear disturbances and fire severity on velvet leaf blueberry abundance, vigor, and berry production in recently burned jack pine forests. Forests, 8, 1–14. 10.3390/f8100398

[jane13130-bib-0016] De Knegt, H. J. , Hengeveld, G. M. , Langevelde, F. V. , De Boer, W. F. , & Kirkman, K. P. (2007). Patch density determines movement patterns and foraging efficiency of large herbivores. Behavioral Ecology, 3, 1065–1072. 10.1093/beheco/arm080

[jane13130-bib-0017] DeMars, C. A. , & Boutin, S. (2017). Nowhere to hide: Effects of linear features on predator‐prey dynamics in a large mammal system. Journal of Animal Ecology, 87, 274–284. 10.1111/1365-2656.12760 28940254

[jane13130-bib-0018] Dickie, M. (2015). The use of anthropogenic linear features by wolves in northeastern Alberta. University of Alberta. MSc Thesis, University of Alberta, Edmonton, Alberta.

[jane13130-bib-0019] Dickie, M. , McNay, R. S. , Sutherland, G. D. , Cody, M. , & Avgar, T. (2019). Data from: Corridors or risk? Movement along, and use of, linear features varies predictably among large mammal predator and prey species. Dryad Digital Repository, 10.5061/dryad.pnvx0k6h8 PMC7028095 31648375

[jane13130-bib-0020] Dickie, M. , Serrouya, R. , Demars, C. , Cranston, J. , & Boutin, S. (2017). Evaluating functional recovery of habitat for threatened woodland caribou. Ecosphere, 8(9), e01936 10.1002/ecs2.1936

[jane13130-bib-0021] Dickie, M. , Serrouya, R. , McNay, R. S. , & Boutin, S. (2017). Faster and farther: Wolf movement on linear features and implications for hunting behaviour. Journal of Applied Ecology, 53, 253–263. 10.1111/1365-2664.12732

[jane13130-bib-0022] Droghini, A. , & Boutin, S. (2017). Snow conditions influence grey wolf (*Canis lupus*) travel paths: The effect of human‐created linear features. Canadian Journal of Zoology, 47, 39–47. 10.1139/cjz-2017-0041

[jane13130-bib-0023] Dyer, S. J. , O’Neill, J. P. , Wasel, S. M. , & Boutin, S. (2002). Quantifying barrier effects of roads and seismic lines on movements of female woodland caribou in northeastern Alberta. Canadian Journal of Zoology, 80(5), 839–845. 10.1139/z02-060

[jane13130-bib-0024] Fahrig, L. (2003). Effects of habitat fragmentation on biodiversity. Annual Review of Ecology, Evolution, and Systematics, 34(1), 487–515. 10.1146/annurev.ecolsys.34.011802.132419

[jane13130-bib-0025] Fahrig, L. , & Rytwinski, T. (2009). Effects of roads on animal abundance : An empirical review and synthesis. Ecology and Society, 14(1), 21.

[jane13130-bib-0026] Finnegan, L. , MacNearney, D. , & Pigeon, K. E. (2018). Divergent patterns of understory forage growth after seismic line exploration: Implications for caribou habitat restoration. Forest Ecology and Management, 409, 634–652. 10.1016/j.foreco.2017.12.010

[jane13130-bib-0027] Fisher, J. T. , & Burton, A. C. (2018). Wildlife winners and losers in an oil sands landscape. Frontiers in Ecology and the Environment, 16(6), 323–328. 10.1002/fee.1807

[jane13130-bib-0028] Fortin, D. , Beyer, H. L. , Boyce, M. S. , Smith, D. W. , Duchesne, T. , & Mao, J. S. (2005). Wolves infleunce elk movements: Behaviour shapes a trophic cascade in Yellowstone National Park. Ecology, 86, 1320–1330.

[jane13130-bib-0029] Frair, J. L. , Merrill, E. H. , Visscher, D. R. , Fortin, D. , Beyer, H. L. , & Morales, J. M. (2005). Scales of movement by elk (*Cervus elaphus*) in response to heterogeneity in forage resources and predation risk. Landscape Ecology, 20(3), 273–287. 10.1007/s10980-005-2075-8

[jane13130-bib-0030] Fryxell, J. M. , Mosser, A. , Sinclair, A. R. E. , & Packer, C. (2007). Group formation stabilizes predator‐prey dynamics. Nature, 449(25), 1041–1044. 10.1038/nature06177 17960242

[jane13130-bib-0031] Fuller, T. (1991). Effect of snow depth on wolf activity and prey selection in north central Minnesota. Canadian Journal of Zoology, 69, 283–287. 10.1139/z91-044

[jane13130-bib-0032] Hervieux, D. , Hebblewhite, M. , DeCesare, N. J. , Russell, M. , Smith, K. , Robertson, S. , & Boutin, S. (2013). Widespread declines in woodland caribou (*Rangifer tarandus caribou*) continue in Alberta. Canadian Journal of Zoology, 91, 872–882.

[jane13130-bib-0033] Holling, C. S. (1959). Some characteristics of simple types of predation and parasitism. The Canadian Entomologist, XCI(7), 385–398. 10.4039/Ent91385-7

[jane13130-bib-0034] Holt, D. (1977). Predation, apparent competition, and the structure of prey communities. Theoretical Population Biology, 12, 197–229. 10.1016/0040-5809(77)90042-9 929457

[jane13130-bib-0035] Houle, M. , Fortin, D. , Dussault, C. , Courtois, R. , & Ouellet, J. P. (2010). Cumulative effects of forestry on habitat use by gray wolf (*Canis lupus*) in the boreal forest. Landscape Ecology, 25(3), 419–433. 10.1007/s10980-009-9420-2

[jane13130-bib-0036] Hutchinson, J. M. C. , & Waser, P. M. (2007). Use, misuse and extensions of 'ideal gas' models of animal encounter. Biological Reviews, 82(3), 335–359. 10.1111/j.1469-185X.2007.00014.x 17624958

[jane13130-bib-0037] James, A. , Boutin, S. , Hebert, D. , & Rippin, A. (2004). Spatial separation of caribou from moose and its relation to predation by wolves. The Journal of Wildlife Management, 68(4), 799–809. 10.2193/0022-541X(2004)068[0799:SSOCFM]2.0.CO;2

[jane13130-bib-0038] James, A. , & Stuart‐Smith, A. (2000). Distribution of caribou and wolves in relation to linear corridors. The Journal of Wildlife Management, 64(1), 154–159. 10.2307/3802985

[jane13130-bib-0039] Johnson, D. H. (1980). The comparison of usage and availability measurements for evaluating resource preference. Ecology, 61(1), 65–71. 10.2307/1937156

[jane13130-bib-0040] Kareiva, P. (1987). Habitat fragmentation and the stability of predator‐prey interactions. Nature, 326, 388–390. 10.1038/330251a0

[jane13130-bib-0041] Kauffman, M. J. , Varley, N. , Smith, D. W. , Stahler, D. R. , MacNulty, D. R. , & Boyce, M. S. (2007). Landscape heterogeneity shapes predation in a newly restored predator‐prey system. Ecology Letters, 10, 690–700. 10.1111/j.1461-0248.2007.01059.x 17594424

[jane13130-bib-0042] Latham, A. , Latham, M. , Boyce, M. S. , & Boutin, S. (2011). Movement responses by wolves to industrial linear features and their effect on woodland caribou in northeastern Alberta. Ecological Applications, 21(8), 2854–2865. 10.1890/11-0666.1

[jane13130-bib-0043] Latham, A. , Latham, M. C. , McCutchen, N. A. , & Boutin, S. (2011). Invading white‐tailed deer change wolf‐caribou dynamics in northeastern Alberta. The Journal of Wildlife Management, 75(1), 204–212. 10.1002/jwmg.28

[jane13130-bib-0044] Lyons, A. L. , Gaines, W. L. , & Servheen, C. (2003). Black bear resource selection in the northeast Cascades, Washington. Biological Conservation, 113(1), 55–62. 10.1016/S0006-3207(02)00349-X

[jane13130-bib-0045] MacCracken, J. G. , Ballenberghe, V. V. , & Peek, J. M. (1993). Use of aquatic plants by moose: Sodium hunger or foraging efficiency? Canadian Journal of Zoology, 71(12), 2345–2351. 10.1139/z93-329

[jane13130-bib-0046] McKenzie, H. , Merrill, E. , Spiteri, R. , & Lewis, M. (2012). How linear features alter predator movement and the functional response. Interface Focus, 2, 205–216. 10.1098/rsfs.2011.0086 22419990 PMC3293201

[jane13130-bib-0047] McLoughlin, P. , Dzus, E. , Wynes, B. , & Boutin, S. (2003). Declines in populations of woodland caribou. The Journal of Wildlife Management, 67(4), 755–761. 10.2307/3802682

[jane13130-bib-0048] Muhly, T. , Semeniuk, C. , Massolo, A. , Hickman, L. , & Musiani, M. (2011). Human activity helps prey win the predator‐prey space race. PLoS ONE, 6(3), e17050 10.1371/journal.pone.0017050 21399682 PMC3047538

[jane13130-bib-0049] Mumma, M. A. , Gillingham, M. P. , Johnson, C. J. , & Parker, K. L. (2017). Understanding predation risk and individual variation in risk avoidance for threatened boreal caribou. Ecology and Evolution, 7, 10266–10277. 10.1002/ece3.3563 29238553 PMC5723594

[jane13130-bib-0050] Murtaugh, P. A. (2007). Simplicity and complexity in ecological data analysis. Ecology, 88(1), 56–62. 10.1890/0012-9658(2007)88[56:SACIED]2.0.CO;2 17489454

[jane13130-bib-0051] Mysterud, A. , & Ims, R. (1998). Functional responses in habitat use: Availability influences relative use in trade‐off situations. Ecology, 79(4), 1435–1441. 10.1890/0012-9658(1998)079[1435:FRIHUA]2.0.CO;2

[jane13130-bib-0052] Newton, E. J. , Patterson, B. R. , Anderson, M. L. , Rodgers, A. R. , Vander Vennen, L. M. , & Fryxell, J. M. (2017). Compensatory selection for roads over natural linear features by wolves in northern Ontario: Implications for caribou conservation. PLoS ONE, 12(11), 1–21. 10.1371/journal.pone.0186525 PMC5695599 29117234

[jane13130-bib-0053] Ofstad, E. , Herfinal, I. , Solberg, E. , & Saether, B. (2016). Home ranges, habitat and body mass: simple correlates of home range size in ungulates. Proceedings of the Royal Society B, 283, 1–8. 10.1098/rspb.2016.1234 PMC5204158 28003441

[jane13130-bib-0054] Osko, T. J. , Hiltz, M. N. , Hudson, R. J. , & Wasel, S. (2004). Moose habitat preferences in response to changing availability. Journal of Wildlife Management, 68(3), 576–584. 10.2193/0022-541X(2004)068[0576:MHPIRT]2.0.CO;2

[jane13130-bib-0055] Prokopenko, C. M. , Boyce, M. S. , & Avgar, T. (2016). Characterizing wildlife behavioural responses to roads using integrated step selection analysis. Journal of Applied Ecology, 54, 470–479. 10.1111/1365-2664.12768

[jane13130-bib-0056] Rempel, R. , Elkie, P. , Rodgers, A. , & Gluck, M. (1997). Timber‐management and natural‐disturbance effects on moose habitat: Landscape evaluation. The Journal of Wildlife Management, 61(2), 517–524. 10.2307/3802610

[jane13130-bib-0057] Rosenzweig, M. L. (1991). Habitat selection and population interactions: The search for mechanism. The American Naturalist, 137, S5–S28. 10.1086/285137

[jane13130-bib-0058] Schick, R. S. , Loarie, S. R. , Colchero, F. , Best, B. D. , Boustany, A. , Conde, D. A. , … Clark, J. S. (2008). Understanding movement data and movement processes: Current and emerging directions. Ecology Letters, 11(12), 1338–1350. 10.1111/j.1461-0248.2008.01249.x 19046362

[jane13130-bib-0059] Schneider, R. R. , & Wasel, S. (2000). The effect of human settlement on the density of moose in northern Alberta. The Journal of Wildlife Management, 64(2), 513–520. 10.2307/3803249

[jane13130-bib-0060] Scrafford, M. A. , Avgar, T. , Heeres, R. , & Boyce, M. S. (2018). Roads elicit negative movement and habitat‐selection responses by wolverines (*Gulo gulo luscus*). Behavioral Ecology, 29(3), 534–542. 10.1093/beheco/arx182

[jane13130-bib-0061] Sorensen, T. , McLoughlin, P. D. , Hervieux, D. , Dzus, E. , Nolan, J. , Wynes, B. , & Boutin, S. (2008). Determining sustainable levels of cumulative effects for boreal caribou. Journal of Wildlife Management, 72(4), 900–905. 10.2193/2007-079

[jane13130-bib-0062] Theuerkauf, J. , Jedrzejewski, W. , Schmidt, K. , & Gula, R. (2003). Spatiotemporal segregation of wolves from humans in the Bialowieza Forest (Poland). The Journal of Wildlife Management, 67(4), 706–716. 10.2307/3802677

[jane13130-bib-0063] Tigner, J. , Bayne, E. M. , & Boutin, S. (2014). Black bear use of seismic lines in Northern Canada. The Journal of Wildlife Management, 78(2), 282–292. 10.1002/jwmg.664

[jane13130-bib-0064] Viana, D. S. , Granados, J. E. , Fandos, P. , Pérez, J. M. , Cano‐Manuel, F. J. , Burón, D. , … Soriguer, R. C. (2018). Linking seasonal home range size with habitat selection and movement in a mountain ungulate. Movement Ecology, 6(1), 1–11. 10.1186/s40462-017-0119-8 29318021 PMC5755340

[jane13130-bib-0065] Vors, L. S. , Schaefer, J. A. , Pond, B. A. , Rodgers, A. , & Patterson, B. (2007). Woodland caribou extirpation and anthropogenic landscape disturbance in Ontario. Journal of Wildlife Management, 71(4), 1249–1256. 10.2193/2006-263

[jane13130-bib-0066] Zimmermann, B. , Nelson, L. , Wabakken, P. , Sand, H. , & Liberg, O. (2014). Behavioral responses of wolves to roads: Scale‐dependent ambivalence. Behavioral Ecology, 25, 1353–1364. 10.1093/beheco/aru134 25419085 PMC4235582

