# Supplementary material for: Corridors or risk? Movement along, and use of, linear features varies predictably among large mammal predator and prey species
Source: J Anim Ecol. 2019 Dec 4;89(2):623–34. doi: 10.1111/1365-2656.13130 (PMC7028095; doi:10.1111/1365-2656.13130)
Supplement: Supplementary file 1 [file JANE-89-623-s001.docx]

# **Appendix S1** – Selection of landcover

Moose on average selected wetlands but avoided deciduous and mixedwood stands (Table 1, Figure S1.1). But most individuals were indifferent to each landcover type (Appendix S7). Individual caribou showed no discernable trends in their response to landcover, though two of four individuals appeared to select coniferous stands (Appendix S7).

Bears on average selected deciduous and mixedwood stands (Table 2, Figure S1.1), a consistent response across 44% of the individuals (Appendix S7). Only 6% of the individuals avoided deciduous and mixedwood stands. Bears were on average indifferent to coniferous stands and avoided wetlands (Table 2), with most individuals having CIs overlapping zero and few individuals selecting either landcover type (Appendix S7). Bears were 1.45 times more likely to select deciduous and mixedwood stands than wetlands.

Wolves on average selected coniferous stands, deciduous and mixedwood stands and wetlands (Table 2, Figure S1.1). Wolves were 1.14 times more likely to select coniferous stands over wetlands. There was consistency among individual wolves, with few individuals avoiding each landcover type (Appendix S7).

We found differences in selection for broad landcover categories between predators and prey. Both wolves and bears selected deciduous and mixedwood stands more than they did wetlands, whereas moose avoided deciduous and mixedwood stands but selected wetlands**.** While all species were predominantly found within wetlands habitats (i.e. the largest proportion of their used locations), reflecting the overwhelmingly large areas occupied by peatlands in Canada’s northern boreal forests, wetland use by predators was much lower than use by prey. This strategy is well documented for woodland caribou, for which an anti-predator strategy appears to be to “space-away” from predators and other prey species (James & Stuart-Smith, 2000). But if moose are also selecting these habitats, at least in this area, this strategy may be less effective in reducing predation rates on caribou. Additional research should focus on understanding how increased human disturbance and natural disturbances are changing spatial overlap and encounters between wolves, bears, their primary prey and caribou across a landscape gradient (but see DeMars and Boutin 2017).


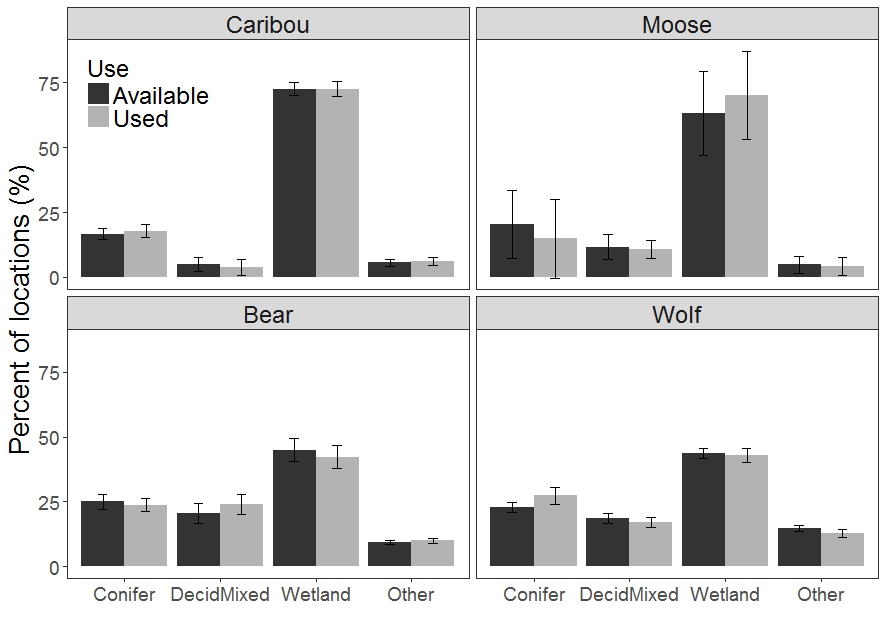


**Figure S1.1**: Mean percent of used and available locations (± SE) within each landcover category. For each species we calculated the percent of locations for each individual separately, and averaged across individuals. Conifer = Coniferous stands; DecidMixed = Deciduous and Mixedwood stands; Wetland = Wetlands; Other = Non-vegetated and unclassified landcover.

# **Appendix S2** – Classifying Riparian Habitat

Unlike anthropogenic LFs, riparian features are often associated with banks and may have game trails along the edges, instead of directly on the features. Therefore, we evaluated the effect of buffer distance on classifying locations as on riparian features (lake shores, streams and rivers). We first classified start and end points as on or off riparian areas at 10, 20, 30, 40, 60, 80, 100, 150 and 200 m. For each buffer width, we then classified each step as on a riparian feature if both the start and end location was classified as on riparian features. We calculated the proportion of steps that were classified as on riparian features using a 200-m buffer, as a function of the buffer width. The majority of steps that were classified as on riparian features at 200 m were classified as on riparian features once the buffer width reached 100 m (Figure S2.1). While wolves did not plateau (Figure S2.1), we chose 100 m as a buffer width to reflect the majority of species.


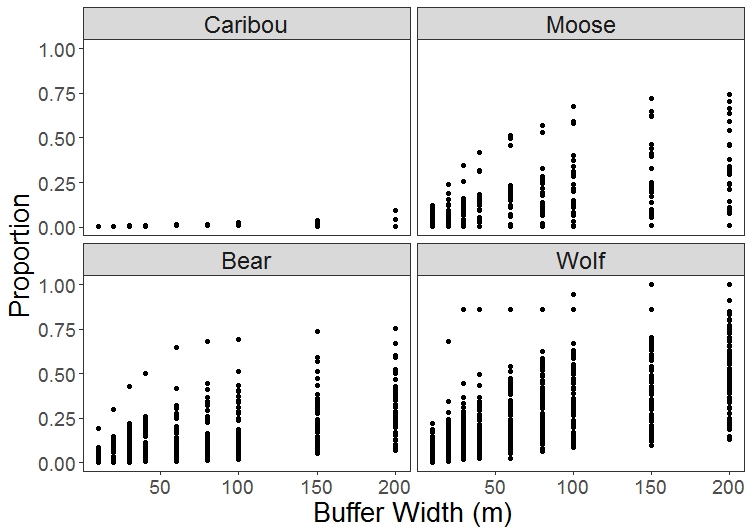


**Figure S2.1:** The proportion of locations, for each species, classified as on riparian features (lake shores, rivers and streams) as a function of the distance to the riparian features. Each data point represents an individual. The proportion of locations classified as on riparian features tends to plateau after the buffer width reaches 100 m.

# **Appendix S3** – Calculating Effect Sizes

To understand how strongly habitat features influenced selection, the relative selection strength was calculated (Avgar, Lele, Keim, & Boyce, 2017). The relative selection of one land cover type over another was calculated as:

$RSS=exp[\beta\left( Habitat1 \right)- \beta(Habitat2)]$. The relative selection strength for anthropogenic disturbances and riparian areas was calculated as the expected tendency of moving towards a given feature type compared to away from it:

$RSS={[\frac{(starting distance+step length)}{(starting distance-step length)}]}^{\beta(Distance to feauture)}$.

Conceptually, this is the ratio of distance to the features if it moved away to distance from feature if it moved towards the disturbance, exponentiated by the selection coefficient (Avgar et al 2017). The calculation is repeated for various starting distances from the habitat type of interest, to evaluate the probability of moving towards vs away from that habitat type as a function of distance.

To understand how strongly habitat features influenced animal movement rates irrespective of habitat selection, the expected displacement rates was calculated for each individual. This was done by numerically integrating over all combinations of step-length and turn-angle values (within a range of {1,10^5^} for step lengths and {0,π} for turn angles) for each species-habitat combination. For each step-length value, the concentration parameter of the von-Mises distribution of turn angles was calculated as:

$$k\left( ln Step length \right)=k_{0}+\beta\left( cos Turn angle \right)+\beta\left( cos Turn angle:ln Step length \right)\cdot ln Step length$$

where $k_{0}$ is the basal (initially estimated) concentration parameter, and $\beta\left( cos TurnAngle \right)$ and $\beta\left( cos Turn angle:ln Step length \right)$ are the iSSA coefficients for the cosine of the turn angle, and the interaction between the cosine of the turn angle and the natural logarithm of the step length, respectively. Similarly, for each turn-angle value, the shape parameter of the Gamma distribution of step lengths was calculated as:

$$s\left( cos Turn angle,habitat \right)=s_{0}+\beta\left( ln Step length \right)+\beta\left( ln Step length:habitat \right)+\beta\left( cos Turn angle:ln Step length \right)\cdot cos Turn angle$$

where $s_{0}$ is the basal (initially estimated) shape parameter, and $\beta\left( ln Step length \right)$ and $\beta\left( ln Step length:habitat \right)$ are the iSSA coefficients for the natural logarithm of the step length, and the interaction between the natural logarithm of the step length and the habitat type, respectively. The joint likelihood (up to a normalization constant) of each step-length - turn-angle combination was then calculated as the product of the probability densities of the value-specific von-Mises and Gamma distributions, and used as weights to calculate the weighted average step length. A simple mean and standard error was then calculated for each species. This approach allows the basal movement parameters and the deviation from that based on model covariates to be incorporated for each individual before averaging.

# **Appendix S4**: The influence of sex on selection and movement responses to human disturbances and natural habitat

Many species exhibit sex- and reproductive status- dependent habitat selection and movement behavior. Males typically have larger home range than females, placing an emphasis on movement corridors for males (Lindzey & Meslow, 1977; Ofstad, Herfinal, Solberg, & Saether, 2016). Females with calves may respond to habitats perceived as riskier than those without calves (DeMars & Boutin, 2017; Mumma, Gillingham, Johnson, & Parker, 2017). Movement-based models are increasingly being developed and used to detect parturition events (Demars, Auger-Methe, Schlagel, & Boutin, 2013; Severud et al., 2015). However, in the absence of ground-truth data to test the accuracy of these models in new systems, the effect of reproductive status on habitat selection and movement patterns is difficult to understand. Furthermore, while movement-based models are fairly effective at detecting parturition, they are less effective at estimating survival of calves, specifically for moose which can have multiple calves (Severud et al., 2015) and are therefore less suited to effectively determine the maternal status after calving. Conversely, the sex of a given individual is recorded during capture and is more reliably estimated. Given sex-dependent differences in space-use and movement behavior, we evaluated the influence of sex on the selection and movement responses to human disturbance and natural habitat. We however lacked the data needed to effectively evaluate the influence of reproductive status.

We evaluated if the selection for, and movement on, each of our habitat attributes of interest depended on if the animal was male or female. We conducted separate models for each species, and upon population-level averaging included sex as a category to determine if the population-level average significantly depended on sex (defined as 95 % Confidence Intervals not overlapping zero). We were only able to test sex-dependent responses for bears and wolves because all monitored caribou and moose were females.

We found no evidence that the selection for anthropogenic LFs, polygonal disturbance or riparian areas for bears and wolves differed between males and females (Table 1). Likewise, animal movement on any of the habitat types of interest did not differ between males and females (Table S4.1). We found sex-dependent differences in basal animal movement, as signified by differences in the logarithmic steplength, cosine of the turnangle, and their interaction (Table S4.1).

Our results support that male and female bears and wolves differed in their basal movement behavior, but these differences do not translate to a difference in selection for, or movement on, human disturbances or natural disturbances in our system. Features that are used as movement corridors such as anthropogenic LFs or riparian areas were selected for and increased movement rates for both males and females to a similar degree.

We were unable to test for the effect of reproductive status in determinizing individual-level variation in responses to these features, or the differences in the response to features pre- and post-reproduction. However, other studies have shown that female caribou with calves avoid anthropogenic LFs more strongly than those without calves (Mumma et al., 2017). While most individual caribou in our study showed insignificant response to anthropogenic LFs, it is possible that the one individual that avoided these features had a calf. Whether reproductive status is an important predictor of individual differences in habitat selection and movement on the human disturbances and natural habitat studied here is an important avenue of future research.

**Table S4.1**: The effect of sex on the average response of bears and wolves to each habitat feature of interest. Individuals were modelled separately and averaged across species with sex as a factor. Female is the reference category. All caribou and moose individuals were female, and only male wolves had estimates for low-impact seismic.

| **Species** | **Component** | **Variable** | **Coefficient** | **SE** |
| --- | --- | --- | --- | --- |
| **Bear** | Selection | Conifer | 0.205 | 0.130 |
|  |  | Deciduous/Mixedwood | -0.140 | 0.150 |
|  |  | Wetland | 0.191 | 0.138 |
|  |  | ln(Distance to LF) | -0.035 | 0.020 |
|  |  | ln(Distance to Poly) | -0.027 | 0.030 |
|  |  | ln(Distance to RIP) | 0.001 | 0.038 |
|  | Movement | ln(SL) | 0.079 | 0.067 |
|  |  | ln(SL):Cos(Turn angle) | 0.043 | 0.016 |
|  |  | Cos(Turn angle) | -0.543 | 0.138 |
|  |  | CLI:ln(SL) | 0.054 | 0.045 |
|  |  | LIS:ln(SL) | - | - |
|  |  | PT:ln(SL) | -0.002 | 0.074 |
|  |  | Poly:ln(SL) | 0.032 | 0.030 |
|  |  | RIP:ln(SL) | -0.012 | 0.020 |
| **Wolf** | Selection | Conifer | 0.058 | 0.122 |
|  |  | Deciduous/Mixedwood | -0.041 | 0.126 |
|  |  | Wetland | -0.052 | 0.086 |
|  |  | ln(Distance to LF) | -0.024 | 0.018 |
|  |  | ln(Distance to Poly) | -0.042 | 0.037 |
|  |  | ln(Distance to RIP) | -0.057 | 0.037 |
|  | Movement | ln(SL) | 0.037 | 0.024 |
|  |  | ln(SL):Cos(Turn angle) | -0.002 | 0.011 |
|  |  | Cos(Turn angle) | -0.081 | 0.153 |
|  |  | CLI:ln(SL) | 0.015 | 0.048 |
|  |  | LIS:ln(SL) | - | - |
|  |  | PT:ln(SL) | 0.024 | 0.051 |
|  |  | Poly:ln(SL) | -0.023 | 0.040 |
|  |  | RIP:ln(SL) | -0.010 | 0.022 |

# **Appendix S5**: Evaluating the influence of time of day, as a proxy for human activity, on animal responses to disturbance

How animals interact with their habitat is expected to differ throughout the day. Crepuscular animals may be more active during twilight hours and more sedentary during the day when temperatures are high (Frair et al., 2005; Prokopenko, Boyce, & Avgar, 2016), placing an emphasis on movement corridors in times of higher movement rates. Furthermore, areas occupied by humans may be perceived as riskier during the day when humans are more active (Muhly, Semeniuk, Massolo, Hickman, & Musiani, 2011; Theuerkauf, Jedrzejewski, Schmidt, & Gula, 2003; Zimmermann, Nelson, Wabakken, Sand, & Liberg, 2014). Given temporal activity patterns of the species monitored, as well as by humans within the system, place varying emphasis on the selection of, and movement in, various habitat types, we conducted a sensitivity analysis to determine if the habitat associations observed in our study were dependent on time of day.

To account for differences in habitat selection and movement patterns associated with high and low human activity, we evaluated if the selection for, and movement on, each of our habitat attributes of interest depended on if it was day or night (Northrup et al., 2012; Zimmermann et al., 2014). We used day and night as proxies for human activity in the absence of direct measurements of vehicle traffic or human activity at facilities, and because differences between high-grade roads and low-grade roads was already accounted for by modelling these features separately. We defined day as sunrise to dusk (one hour before sunset), and night as dusk (one hour before sunrise) to sunrise.

We conducted separate models for each species and time of day category, and upon population-level averaging included day vs. night as a category to determine if the population-level average significantly (defined as 95 % Confidence Intervals not overlapping zero) depended on time of day. We also calculated the average selection for anthropogenic LFs, polygonal disturbances and riparian areas, as well as the expected speeds in each feature type of interest for each species, time of day combination.

We found that the average selection for anthropogenic LFs, polygonal disturbance and riparian areas for each species typically did not differ significantly between day and night (Table S5.1, S5.2). However, wolves selected to be closer to anthropogenic LFs during the day, and this response was stronger at night (Table S5.2). Likewise, each species typically did not respond to each feature of interest differently in the day than at night. Moose and wolves moved faster on conventional seismic, low-impact roads and ice roads in the day, and this effect was significantly stronger during the night (Table S5.1, S5.2). In general, animals have higher expected speeds at night than during the day.

Our results show that our inferences on behavioural responses to habitat attributes of interest, particularly human disturbances and riparian areas, is not sensitive to the time of day. In no cases did time of day change the direction of selection or movement response. When there was a significant effect of time of day, animals typically strengthened their response to a feature during the night. Wolves in particular selected anthropogenic LFs more strongly at night and moved faster while on them. Given the response to anthropogenic LFs was enhanced during the night when humans are less active, this may suggest that wolves perceive human activity on anthropogenic LFs to be risky (Zimmermann et al., 2014). However, given wolves still selected for, and moved faster on anthropogenic LFs during the day suggests that the benefits of movement enhancement outweigh this cost. This may not be true in areas with higher human activity, or in areas where there is increased hunting pressure (Muhly et al., 2011; Whittington, St. Clair, & Mercer, 2005).

**Table S5.1**: Average prey selection and movement responses to human disturbances and natural habitat during day and night. Each individual was modelled separately then averaged using inverse variance models by species and time of day for each parameter of interest. CLI = Conventional seismic, low-grade roads and ice-roads, LIS = low-impact seismic, PT = pipelines and transmission lines, Poly = polygonal disturbances and RIP = riparian habitat, SL = step length.

| **Species** | **Component** | **Variable** | **Day** | |  | **Night** | |
| --- | --- | --- | --- | --- | --- | --- | --- |
|  |  |  | **Coefficient** | **SE** |  | **Coefficient** | **SE** |
| Caribou | Selection | Conifer | -0.090 | 0.052 |  | 0.089 | 0.443 |
|  |  | Deciduous/Mixedwood | 0.133 | 0.006 |  | -8.591 | 0.344 |
|  |  | Wetland | -1.610 | 0.018 |  | -5.102 | 2.268 |
|  |  | ln(Distance to LF) | -0.047 | 0.208 |  | -0.111 | 0.285 |
|  |  | ln(Distance to Poly) | 1.678 | 1.578 |  | 9.227 | 6.073 |
|  |  | ln(Distance to RIP) | 0.387 | 0.147 |  | 0.415 | 0.507 |
|  | Movement | ln(SL) | -0.502 | - |  | -0.420 | - |
|  |  | ln(SL):Cos(Turn angle) | 0.278 | 0.022 |  | 0.351 | 0.023 |
|  |  | Cos(Turn angle) | -1.277 | 0.100 |  | -1.124 | 0.065 |
|  |  | CLI:ln(SL) | - | - |  | - | - |
|  |  | LIS:ln(SL) | - | - |  | - | - |
|  |  | PT:ln(SL) | - | - |  | - | - |
|  |  | Poly:ln(SL) | - | - |  | - | - |
|  |  | RIP:ln(SL) | 0.134 | 0.041 |  | 0.160 | 0.001 |
| Moose | Selection | Conifer | -0.118 | 0.130 |  | -0.034 | 0.162 |
|  |  | Deciduous/Mixedwood | -0.254 | 0.133 |  | -0.208 | 0.184 |
|  |  | Wetland | 0.285 | 0.140 |  | 0.188 | 0.180 |
|  |  | ln(Distance to LF) | 0.138 | 0.034 |  | 0.091 | 0.048 |
|  |  | ln(Distance to Poly) | -0.299 | 0.397 |  | 0.392 | 0.396 |
|  |  | ln(Distance to RIP) | -0.036 | 0.034 |  | -0.079 | 0.030 |
|  | Movement | ln(SL) | -0.880 | 0.044 |  | -0.881 | 0.031 |
|  |  | ln(SL):Cos(Turn angle) | 0.204 | 0.010 |  | 0.233 | 0.008 |
|  |  | Cos(Turn angle) | -0.840 | 0.050 |  | -0.784 | 0.057 |
|  |  | CLI:ln(SL) | 0.323 | - |  | 0.522 | - |
|  |  | LIS:ln(SL) | - | - |  | - | - |
|  |  | PT:ln(SL) | - | - |  | - | - |
|  |  | Poly:ln(SL) | - | - |  | - | - |
|  |  | RIP:ln(SL) | 0.095 | 0.026 |  | 0.108 | 0.028 |

**Table S5.2**: Average predator selection and movement responses to human disturbances and natural habitat during day and night. Each individual was modelled separately then averaged using inverse variance models by species and time of day for each parameter of interest. CLI = Conventional seismic, low-grade roads and ice-roads, LIS = low-impact seismic, PT = pipelines and transmission lines, Poly = polygonal disturbances and RIP = riparian habitat, SL = step length.

| **Species** | **Component** | **Variable** | **Day** | |  | **Night** | |
| --- | --- | --- | --- | --- | --- | --- | --- |
|  |  |  | **Coefficient** | **SE** |  | **Coefficient** | **SE** |
| **Bear** | Selection | Conifer | -0.097 | 0.080 |  | -0.159 | 0.083 |
|  |  | Deciduous/Mixedwood | 0.256 | 0.109 |  | 0.200 | 0.093 |
|  |  | Wetland | -0.114 | 0.094 |  | -0.242 | 0.078 |
|  |  | ln(Distance to LF) | -0.074 | 0.019 |  | 0.003 | 0.018 |
|  |  | ln(Distance to Poly) | -0.066 | 0.035 |  | -0.100 | 0.055 |
|  |  | ln(Distance to RIP) | -0.037 | 0.025 |  | -0.051 | 0.031 |
|  | Movement | ln(SL) | -0.883 | 0.129 |  | -1.095 | 0.055 |
|  |  | ln(SL):Cos(Turn angle) | 0.642 | 0.010 |  | 0.628 | 0.009 |
|  |  | Cos(Turn angle) | -3.053 | 0.127 |  | -2.959 | 0.115 |
|  |  | CLI:ln(SL) | 0.243 | 0.034 |  | 0.284 | 0.083 |
|  |  | LIS:ln(SL) | - | - |  | - | - |
|  |  | PT:ln(SL) | 0.353 | 0.064 |  | 0.303 | 0.047 |
|  |  | Poly:ln(SL) | 0.117 | 0.031 |  | 0.102 | 0.046 |
|  |  | RIP:ln(SL) | 0.008 | 0.019 |  | -0.002 | 0.013 |
| **Wolf** | Selection | Conifer | 0.296 | 0.059 |  | 0.375 | 0.085 |
|  |  | Deciduous/Mixedwood | 0.304 | 0.059 |  | 0.355 | 0.088 |
|  |  | Wetland | 0.140 | 0.039 |  | 0.187 | 0.071 |
|  |  | ln(Distance to LF) | -0.041 | 0.010 |  | -0.076 | 0.013 |
|  |  | ln(Distance to Poly) | -0.035 | 0.024 |  | -0.073 | 0.023 |
|  |  | ln(Distance to RIP) | -0.048 | 0.021 |  | -0.045 | 0.019 |
|  | Movement | ln(SL) | -0.816 | 0.016 |  | -0.740 | 0.022 |
|  |  | ln(SL):Cos(Turn angle) | 0.551 | 0.005 |  | 0.553 | 0.006 |
|  |  | Cos(Turn angle) | -2.528 | 0.079 |  | -2.587 | 0.089 |
|  |  | CLI:ln(SL) | 0.272 | 0.028 |  | 0.324 | 0.042 |
|  |  | LIS:ln(SL) | 0.106 | - |  | 1.537 | - |
|  |  | PT:ln(SL) | 0.366 | 0.059 |  | 0.384 | 0.124 |
|  |  | Poly:ln(SL) | -0.013 | 0.028 |  | -0.010 | 0.041 |
|  |  | RIP:ln(SL) | 0.090 | 0.010 |  | 0.083 | 0.013 |

**Table S5.3**: Full model output for caribou population-level averaging of selection and movement responses during day and night. CLI = Conventional seismic, low-grade roads and ice-roads, LIS = low-impact seismic, PT = pipelines and transmission lines, Poly = polygonal disturbances and RIP = riparian habitat, SL = step length.

| **Variable** | | **Day** | |  | **Night** | |
| --- | --- | --- | --- | --- | --- | --- |
|  |  | **Coefficient** | **SE** |  | **Coefficient** | **SE** |
| Conifer | Intercept | -0.090 | 0.052 |  | 0.089 | 0.443 |
| Conifer | Percent Conifer | 0.176 | 0.037 |  | 0.049 | 0.310 |
| Deciduous/Mixedwood | Intercept | 0.133 | 0.006 |  | -8.591 | 0.344 |
| Deciduous/Mixedwood | Percent Deciduous /Mixedwood | -0.160 | 0.004 |  | 4.622 | 0.212 |
| Wetland | Intercept | -1.610 | 0.018 |  | -5.102 | 2.268 |
| Wetland | Percent Wetland | 3.102 | 0.035 |  | 10.851 | 4.648 |
| ln(Distance to LF) | Intercept | -0.047 | 0.208 |  | -0.111 | 0.285 |
| ln(Distance to LF) | Mean Available Distance | -0.092 | 0.169 |  | -0.134 | 0.240 |
| ln(Distance to Poly) | Intercept | 1.678 | 1.578 |  | 9.227 | 6.073 |
| ln(Distance to Poly) | Mean Available Distance | 1.283 | 1.500 |  | 14.481 | 7.420 |
| ln(Distance to RIP) | Intercept | 0.387 | 0.147 |  | 0.415 | 0.507 |
| ln(Distance to RIP) | Mean Available Distance | -0.241 | 0.167 |  | 0.237 | 0.491 |
| ln(SL) | Intercept | -0.502 | - |  | -0.420 | - |
| ln(SL) | Cos(TurnAngle) | -0.084 | - |  | -0.520 | - |
| ln(SL) | ln(SL): Cos(TurnAngle) | -0.085 | - |  | -0.433 | - |
| ln(SL) | RIP:ln(SL) | -0.499 | - |  | -0.086 | - |
| ln(SL): Cos(TurnAngle) | Intercept | 0.278 | 0.022 |  | 0.351 | 0.023 |
| ln(SL): Cos(TurnAngle) | Cos(TurnAngle) | -0.074 | 0.020 |  | -0.087 | 0.030 |
| ln(SL): Cos(TurnAngle) | ln(SL) | -0.032 | 0.021 |  | -0.019 | 0.019 |
| Cos(TurnAngle) | Intercept | -1.277 | 0.100 |  | -1.124 | 0.065 |
| Cos(TurnAngle) | ln(SL) | -0.020 | 0.111 |  | 0.022 | 0.070 |
| RIP:ln(SL) | Intercept | 0.134 | 0.041 |  | 0.160 | 0.001 |
| RIP:ln(SL) | ln(SL) | -0.111 | 0.072 |  | -0.147 | 0.001 |
| RIP:ln(SL) | Percent Available Steps in RIP | -0.003 | 0.058 |  | 0.028 | 0.001 |

**Table S5.4**: Full model output for moose population-level averaging of selection and movement responses during day and night. CLI = Conventional seismic, low-grade roads and ice-roads, LIS = low-impact seismic, PT = pipelines and transmission lines, Poly = polygonal disturbances and RIP = riparian habitat, SL = step length.

| **Variable** | | **Day** | |  | **Night** | |
| --- | --- | --- | --- | --- | --- | --- |
|  |  | **Coefficient** | **SE** |  | **Coefficient** | **SE** |
| Conifer | Percent Conifer | -0.118 | 0.130 |  | -0.034 | 0.162 |
| Conifer | Intercept | -0.042 | 0.137 |  | -0.093 | 0.202 |
| Deciduous/Mixedwood | Percent Decid/Mixed | -0.254 | 0.133 |  | -0.208 | 0.184 |
| Deciduous/Mixedwood | Intercept | -0.065 | 0.188 |  | -0.208 | 0.291 |
| Wetland | Percent Wetland | 0.285 | 0.140 |  | 0.188 | 0.180 |
| Wetland | Intercept | 0.300 | 0.196 |  | 0.087 | 0.249 |
| ln(Distance to LF) | Intercept | 0.138 | 0.034 |  | 0.091 | 0.048 |
| ln(Distance to LF) | Mean Available Distance | 0.024 | 0.017 |  | 0.033 | 0.025 |
| ln(Distance to Poly) | Intercept | -0.299 | 0.397 |  | 0.392 | 0.396 |
| ln(Distance to Poly) | Mean Available Distance | -0.237 | 0.245 |  | 0.254 | 0.205 |
| ln(Distance to RIP) | Intercept | -0.036 | 0.034 |  | -0.079 | 0.030 |
| ln(Distance to RIP) | Mean Available Distance | -0.009 | 0.047 |  | 0.035 | 0.024 |
| ln(SL) | Intercept | -0.880 | 0.044 |  | -0.881 | 0.031 |
| ln(SL) | Cos(TurnAngle) | -0.215 | 0.106 |  | -0.129 | 0.080 |
| ln(SL) | ln(SL): Cos(TurnAngle) | -0.186 | 0.146 |  | -0.119 | 0.072 |
| ln(SL) | CLI:ln(SL) | 0.332 | 0.373 |  | 0.138 | 0.099 |
| ln(SL) | LIS:ln(SL) | 1.906 | 2.212 |  | 0.150 | 0.119 |
| ln(SL) | Poly:ln(SL) | 0.269 | 0.317 |  | -0.089 | 0.078 |
| ln(SL) | RIP:ln(SL) | 0.083 | 0.052 |  | -0.038 | 0.025 |
| ln(SL): Cos(TurnAngle) | Intercept | 0.204 | 0.010 |  | 0.233 | 0.008 |
| ln(SL): Cos(TurnAngle) | ScaleCosTA | -0.083 | 0.013 |  | -0.099 | 0.008 |
| ln(SL): Cos(TurnAngle) | ln(SL) | -0.012 | 0.012 |  | -0.019 | 0.010 |
| Cos(TurnAngle) | Intercept | -0.840 | 0.050 |  | -0.784 | 0.057 |
| Cos(TurnAngle) | ln(SL) | -0.071 | 0.054 |  | -0.114 | 0.066 |
| CLI:ln(SL) | Intercept | 0.323 | - |  | 0.522 | - |
| CLI:ln(SL) | ln(SL) | 0.356 | - |  | 0.149 | - |
| CLI:ln(SL) | Percent Available Steps in CLI | -0.077 | - |  | -0.110 | - |
| RIP:ln(SL) | Intercept | 0.095 | 0.026 |  | 0.108 | 0.028 |
| RIP:ln(SL) | ln(SL) | 0.019 | 0.028 |  | -0.080 | 0.034 |
| RIP:ln(SL) | Percent Available Steps in RIP | 0.054 | 0.025 |  | 0.058 | 0.029 |

**Table S5.5**: Full model output for bear population-level averaging of selection and movement responses during day and night. CLI = Conventional seismic, low-grade roads and ice-roads, LIS = low-impact seismic, PT = pipelines and transmission lines, Poly = polygonal disturbances and RIP = riparian habitat, SL = step length.

| **Variable** | | **Day** | |  | **Night** | |
| --- | --- | --- | --- | --- | --- | --- |
|  |  | **Coefficient** | **SE** |  | **Coefficient** | **SE** |
| Conifer | Percent Conifer | -0.097 | 0.080 |  | -0.159 | 0.083 |
| Conifer | Intercept | -0.096 | 0.080 |  | -0.030 | 0.077 |
| Deciduous/Mixedwood | Percent Decid/Mixed | 0.256 | 0.109 |  | 0.200 | 0.093 |
| Deciduous/Mixedwood | Intercept | -0.141 | 0.095 |  | -0.164 | 0.078 |
| Wetland | Percent Wetland | -0.114 | 0.094 |  | -0.242 | 0.078 |
| Wetland | Intercept | 0.156 | 0.106 |  | 0.208 | 0.092 |
| ln(Distance to LF) | Intercept | -0.074 | 0.019 |  | 0.003 | 0.018 |
| ln(Distance to LF) | Mean Available Distance | -0.061 | 0.024 |  | 0.032 | 0.020 |
| ln(Distance to Poly) | Intercept | -0.066 | 0.035 |  | -0.100 | 0.055 |
| ln(Distance to Poly) | Mean Available Distance | -0.030 | 0.025 |  | -0.053 | 0.040 |
| ln(Distance to RIP) | Intercept | -0.037 | 0.025 |  | -0.051 | 0.031 |
| ln(Distance to RIP) | Mean Available Distance | 0.126 | 0.033 |  | 0.074 | 0.035 |
| ln(SL) | Intercept | -0.883 | 0.129 |  | -1.095 | 0.055 |
| ln(SL) | Cos(TurnAngle) | 0.289 | 0.378 |  | 0.184 | 0.241 |
| ln(SL) | ln(SL): Cos(TurnAngle) | 0.291 | 0.386 |  | 0.289 | 0.234 |
| ln(SL) | CLI:ln(SL) | 0.020 | 0.130 |  | -0.034 | 0.074 |
| ln(SL) | LIS:ln(SL) | -0.011 | 0.230 |  | 0.150 | 0.105 |
| ln(SL) | PT:ln(SL) | 0.039 | 0.400 |  | 0.324 | 0.202 |
| ln(SL) | Poly:ln(SL) | -0.415 | 0.454 |  | 0.081 | 0.089 |
| ln(SL) | RIP:ln(SL) | -0.018 | 0.110 |  | -0.008 | 0.067 |
| ln(SL): Cos(TurnAngle) | Intercept | 0.642 | 0.010 |  | 0.628 | 0.009 |
| ln(SL): Cos(TurnAngle) | Cos(TurnAngle) | -0.206 | 0.014 |  | -0.220 | 0.012 |
| ln(SL): Cos(TurnAngle) | ln(SL) | 0.024 | 0.013 |  | 0.015 | 0.012 |
| Cos(TurnAngle) | Intercept | -3.053 | 0.127 |  | -2.959 | 0.115 |
| Cos(TurnAngle) | ln(SL) | 0.107 | 0.170 |  | -0.235 | 0.131 |
| CLI:ln(SL) | Intercept | 0.243 | 0.034 |  | 0.284 | 0.083 |
| CLI:ln(SL) | ln(SL) | 0.031 | 0.037 |  | 0.003 | 0.066 |
| CLI:ln(SL) | Percent Available Steps in CLI | -0.036 | 0.023 |  | -0.038 | 0.044 |
| PT:ln(SL) | Intercept | 0.353 | 0.064 |  | 0.303 | 0.047 |
| PT:ln(SL) | ln(SL) | -0.060 | 0.051 |  | 0.001 | 0.042 |
| PT:ln(SL) | Percent Available Steps in PT | 0.006 | 0.045 |  | 0.015 | 0.027 |
| Poly:ln(SL) | Intercept | 0.117 | 0.031 |  | 0.102 | 0.046 |
| Poly:ln(SL) | ln(SL) | -0.039 | 0.027 |  | -0.015 | 0.040 |
| Poly:ln(SL) | Percent Available Steps in Poly | -0.029 | 0.015 |  | -0.026 | 0.017 |
| RIP:ln(SL) | Intercept | 0.008 | 0.019 |  | -0.002 | 0.013 |
| RIP:ln(SL) | ln(SL) | 0.006 | 0.022 |  | -0.052 | 0.021 |
| RIP:ln(SL) | Percent Available Steps in RIP | -0.007 | 0.016 |  | 0.047 | 0.020 |
|  |  |  |  |  |  |  |

**Table S5.6**: Full model output for wolf population-level averaging of selection and movement responses during day and night. CLI = Conventional seismic, low-grade roads and ice-roads, LIS = low-impact seismic, PT = pipelines and transmission lines, Poly = polygonal disturbances and RIP = riparian habitat, SL = step length.

| **Variable** | | **Day** | |  | **Night** | |
| --- | --- | --- | --- | --- | --- | --- |
|  |  | **Coefficient** | **SE** |  | **Coefficient** | **SE** |
| Conifer | Percent Conifer | 0.296 | 0.059 |  | 0.375 | 0.085 |
| Conifer | Intercept | 0.153 | 0.073 |  | 0.329 | 0.106 |
| Deciduous/Mixedwood | Percent Decid/Mixed | 0.304 | 0.059 |  | 0.355 | 0.088 |
| Deciduous/Mixedwood | Intercept | -0.232 | 0.060 |  | -0.336 | 0.081 |
| Wetland | Percent Wetland | 0.140 | 0.039 |  | 0.187 | 0.071 |
| Wetland | Intercept | 0.115 | 0.041 |  | 0.167 | 0.074 |
| ln(Distance to LF) | Intercept | -0.041 | 0.010 |  | -0.076 | 0.013 |
| ln(Distance to LF) | Mean Available Distance | -0.051 | 0.010 |  | -0.042 | 0.013 |
| ln(Distance to Poly) | Intercept | -0.035 | 0.024 |  | -0.073 | 0.023 |
| ln(Distance to Poly) | Mean Available Distance | 0.022 | 0.022 |  | -0.011 | 0.021 |
| ln(Distance to RIP) | Intercept | -0.048 | 0.021 |  | -0.045 | 0.019 |
| ln(Distance to RIP) | Mean Available Distance | -0.036 | 0.023 |  | 0.009 | 0.019 |
| ln(SL) | Intercept | -0.816 | 0.016 |  | -0.740 | 0.022 |
| ln(SL) | Cos(TurnAngle) | -0.170 | 0.057 |  | -0.057 | 0.080 |
| ln(SL) | ln(SL): Cos(TurnAngle) | -0.152 | 0.057 |  | -0.100 | 0.090 |
| ln(SL) | CLI:ln(SL) | -0.037 | 0.033 |  | -0.061 | 0.037 |
| ln(SL) | LIS:ln(SL) | 0.100 | 0.248 |  | 0.001 | 0.052 |
| ln(SL) | PT:ln(SL) | -0.019 | 0.030 |  | 0.000 | 0.033 |
| ln(SL) | Poly:ln(SL) | 0.004 | 0.035 |  | -0.052 | 0.029 |
| ln(SL) | RIP:ln(SL) | -0.038 | 0.020 |  | 0.036 | 0.030 |
| ln(SL): Cos(TurnAngle) | Intercept | 0.551 | 0.005 |  | 0.553 | 0.006 |
| ln(SL): Cos(TurnAngle) | Cos(TurnAngle) | -0.105 | 0.005 |  | -0.097 | 0.005 |
| ln(SL): Cos(TurnAngle) | ln(SL) | -0.027 | 0.007 |  | -0.003 | 0.006 |
| Cos(TurnAngle) | Intercept | -2.528 | 0.079 |  | -2.587 | 0.089 |
| Cos(TurnAngle) | ln(SL) | 0.006 | 0.097 |  | 0.103 | 0.093 |
| CLI:ln(SL) | Intercept | 0.272 | 0.028 |  | 0.324 | 0.042 |
| CLI:ln(SL) | ln(SL) | -0.014 | 0.030 |  | -0.081 | 0.041 |
| CLI:ln(SL) | Percent Available Steps in CLI | -0.005 | 0.025 |  | 0.019 | 0.027 |
| LIS:ln(SL) | Intercept | 0.106 | - |  | 1.537 | - |
| LIS:ln(SL) | ln(SL) | 0.049 | - |  | 2.620 | - |
| LIS:ln(SL) | Percent Available Steps in LIS | 0.002 | - |  | -1.142 | - |
| PT:ln(SL) | Intercept | 0.366 | 0.059 |  | 0.384 | 0.124 |
| PT:ln(SL) | ln(SL) | 0.000 | 0.034 |  | -0.079 | 0.059 |
| PT:ln(SL) | Percent Available Steps in PT | -0.039 | 0.049 |  | -0.029 | 0.077 |
| Poly:ln(SL) | Intercept | -0.013 | 0.028 |  | -0.010 | 0.041 |
| Poly:ln(SL) | ln(SL) | -0.052 | 0.033 |  | -0.027 | 0.038 |
| Poly:ln(SL) | Percent Available Steps in Poly | -0.001 | 0.019 |  | 0.011 | 0.021 |
| RIP:ln(SL) | Intercept | 0.090 | 0.010 |  | 0.083 | 0.013 |
| RIP:ln(SL) | ln(SL) | -0.050 | 0.013 |  | 0.026 | 0.014 |
| RIP:ln(SL) | Percent Available Steps in RIP | -0.043 | 0.011 |  | -0.051 | 0.012 |

**Appendix S6** - Description of habitat use and availability

Data used in this study reflect fine-scale habitat selection and movement, even beyond those typically used to understand movement behavior using GPS data. However, the density of LFs may impact individual habitat selection and movement responses to these features as well as the response to natural LFs (Johnson, 1980; Mysterud & Ims, 1998; Newton et al., 2017). While all animals were captured within the same study area, predator species had substantially larger home ranges than prey species (Figure S6.1). These differences in home range size resulted in moose and caribou with home ranges that tended to be constrained to lower LF-density areas whereas bears and wolves had larger home-ranges that incorporated a variety of disturbance densities (Figure S6.1). As such, the average density of LFs within the composite 100 % Minimum Convex Polygons reflected this pattern (Figure S6.2). The pattern of bears and wolves having higher density LFs within their home range is also reflected in the fine-scale habitat availability generated from the random steps (Table S6.1). Wolves and bears tended to have a higher mean number of available and used steps within disturbance categories than moose and caribou (Tables S6.1).

We additionally present the mean (and standard error) steplength (m) of observed 15-minute steps (Table S6.2) in which both the start and end points were classified within each disturbance category of interest.


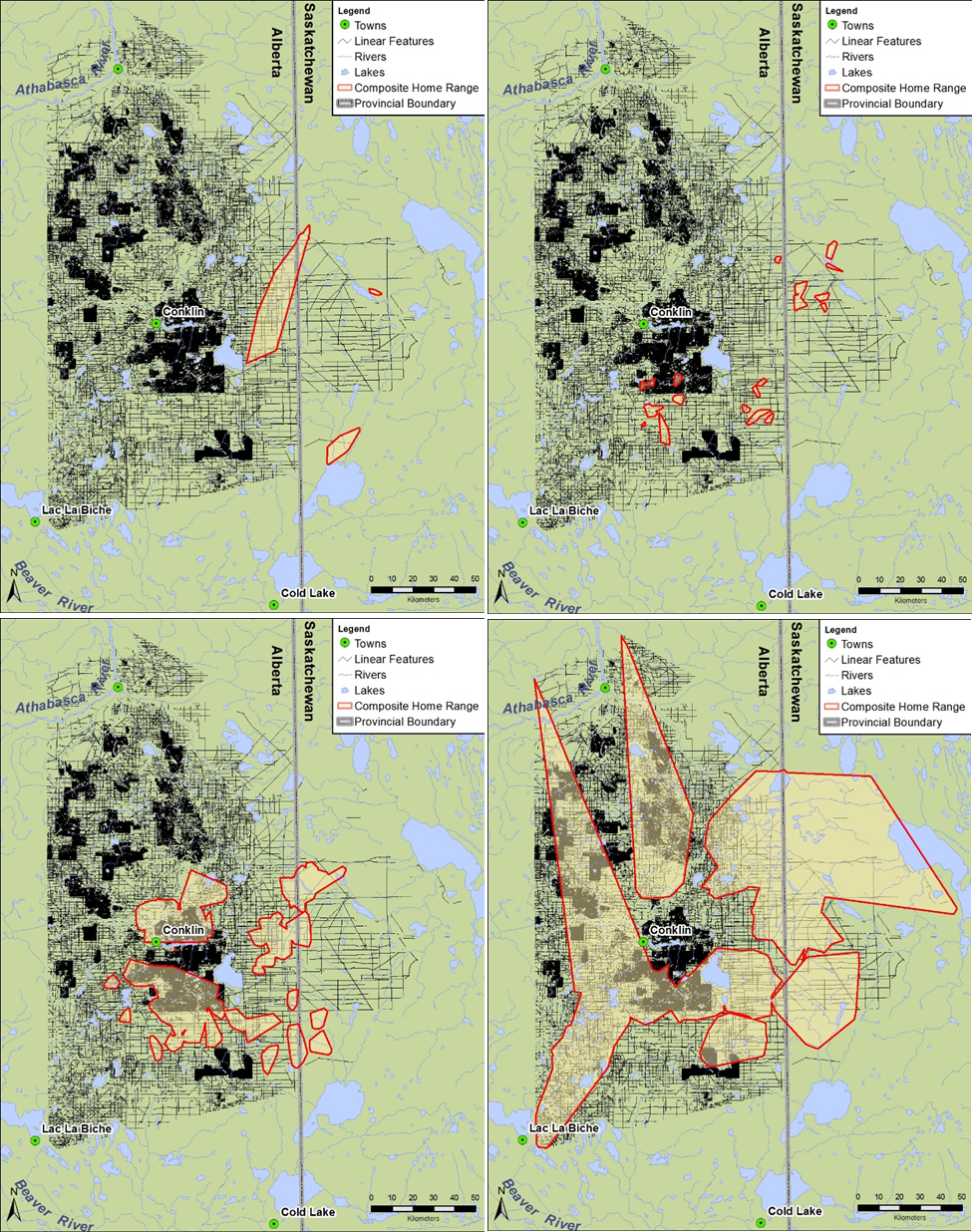


B

D

C

A

**Figure S6.1**: Study area map depicting anthropogenic LFs (black shading), rivers and lakes within the composite 100% Minimum Convex Polygons (red) of collared caribou (A), moose (B), black bears (C) and wolves (D).


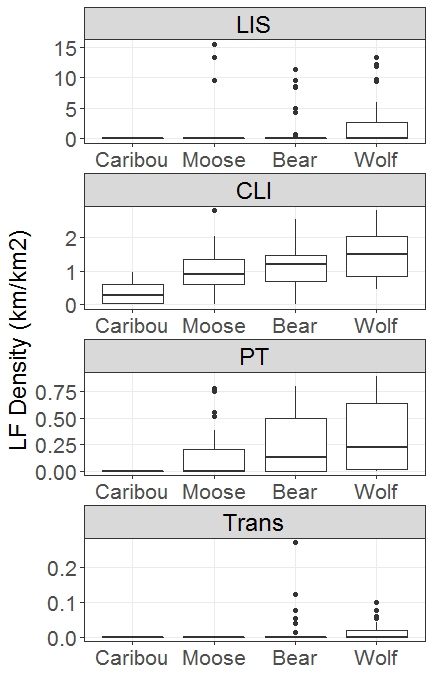


**Figure S6.2**: Density (km/km^2^) of each linear feature class within the composite 100% Minimum Convex Polygons of individual caribou, moose, bears and wolves with 15-minute data. CLI = Conventional seismic, low-grade roads and ice-roads, LIS = low-impact seismic, PT = pipelines and transmission lines, TRAN = railway or high-grade road.

**Table S6.1**: The mean (and standard error) percentage of available and used steps in which both the start and end points were classified within each disturbance category of interest. The percentage of available and used steps in each disturbance type was calculated per individual and averaged across each species. Poly = polygonal disturbances, LIS = low-impact seismic, CLI = Conventional seismic, low-grade roads and ice-roads, PT = pipelines and transmission lines, TRAN = railway or high-grade road, and ND = undisturbed habitat.

| **Species** | **Disturbance** | **Available** | |  | **Used** | |
| --- | --- | --- | --- | --- | --- | --- |
|  |  | **Mean** | **SE** |  | **Mean** | **SE** |
| Caribou | Poly | 0.00 | 1.63 |  | 0.00 | 1.44 |
|  | LIS | 0.00 | 0.05 |  | 0.00 | 0.08 |
|  | CLI | 0.13 | 0.15 |  | 0.29 | 0.38 |
|  | PT | 0.00 | 0.19 |  | 0.00 | 0.47 |
|  | TRAN | 0.00 | 0.01 |  | 0.00 | 0.01 |
|  | ND | 99.87 | 1.83 |  | 99.71 | 1.91 |
| Moose | Poly | 1.08 | 0.73 |  | 0.97 | 0.66 |
|  | LIS | 0.25 | 0.18 |  | 0.38 | 0.31 |
|  | CLI | 0.51 | 0.15 |  | 0.72 | 0.24 |
|  | PT | 0.03 | 0.02 |  | 0.03 | 0.02 |
|  | TRAN | 0.00 | 0.00 |  | 0.00 | 0.00 |
|  | ND | 98.12 | 1.00 |  | 97.89 | 1.11 |
| Bear | Poly | 6.52 | 1.92 |  | 6.02 | 1.69 |
|  | LIS | 0.12 | 0.06 |  | 0.18 | 0.08 |
|  | CLI | 0.98 | 0.17 |  | 2.33 | 0.44 |
|  | PT | 1.04 | 0.22 |  | 2.38 | 0.54 |
|  | TRAN | 0.02 | 0.01 |  | 0.02 | 0.02 |
|  | ND | 91.32 | 2.14 |  | 89.08 | 2.22 |
| Wolf | Poly | 9.26 | 1.98 |  | 7.17 | 1.67 |
|  | LIS | 0.22 | 0.09 |  | 0.58 | 0.24 |
|  | CLI | 1.58 | 0.20 |  | 4.69 | 0.73 |
|  | PT | 0.85 | 0.16 |  | 1.73 | 0.35 |
|  | TRAN | 0.10 | 0.04 |  | 0.24 | 0.08 |
|  | ND | 87.99 | 2.22 |  | 85.59 | 2.38 |

**Table S6.2**: The mean (and standard error) steplength (m) of observed 15-minute steps in which both the start and end points were classified within each disturbance category of interest. The mean steplength in each disturbance type was calculated per individual, and averaged across each species. Poly = polygonal disturbances, LIS = low-impact seismic, CLI = Conventional seismic, low-grade roads and ice-roads, PT = pipelines and transmission lines, TRAN = railway or high-grade road, and ND = undisturbed habitat.

| **Species** | **Disturbance** | **Mean** | **SE** |
| --- | --- | --- | --- |
| Caribou | Poly | - | - |
|  | LIS | - | - |
|  | CLI | 314.49 | 310.88 |
|  | PT | - | - |
|  | TRAN | - | - |
|  | ND | 53.33 | 4.53 |
| Moose | Poly | 141.70 | 90.50 |
|  | LIS | 31.93 | 19.16 |
|  | CLI | 107.63 | 63.93 |
|  | PT | 344.64 | 196.15 |
|  | TRAN | - | - |
|  | ND | 25.90 | 1.50 |
| Bear | Poly | 138.37 | 14.90 |
|  | LIS | 187.84 | 37.04 |
|  | CLI | 198.18 | 21.64 |
|  | PT | 199.45 | 27.17 |
|  | TRAN | 230.98 | 52.03 |
|  | ND | 108.45 | 5.48 |
| Wolf | Poly | 421.29 | 64.67 |
|  | LIS | 107.15 | 25.33 |
|  | CLI | 639.76 | 75.80 |
|  | PT | 813.12 | 100.54 |
|  | TRAN | 797.77 | 104.29 |
|  | ND | 202.91 | 9.07 |

**Appendix S7** – Individual Selection and Movement Responses

**Table S7.1**: Prey individual responses to human disturbances and natural habitat. Negative and positive are defined as model coefficients with 95% CIs that do not overlap zero, and neutral as model coefficients with 95% CIs that overlap zero. CLI = Conventional seismic, low-grade roads and ice-roads, LIS = low-impact seismic, PT = pipelines and transmission lines, Poly = polygonal disturbances and RIP = riparian habitat.

| **Species** | **Component** | **Variable** |  | **Response** | | | |
| --- | --- | --- | --- | --- | --- | --- | --- |
|  |  |  |  | **-** | **+** | **=** | **n** |
| Caribou | Selection | Conifer |  | 1 | 2 | 1 | 4 |
|  |  | Deciduous/Mixedwood |  | 1 | 0 | 3 | 4 |
|  |  | Wetland |  | 0 | 1 | 2 | 3 |
|  |  | ln(Distance to LF) |  | 0 | 1 | 3 | 4 |
|  |  | ln(Distance to Poly) |  | 0 | 1 | 3 | 4 |
|  |  | ln (Distance to RIP) |  | 0 | 2 | 2 | 4 |
|  | Movement | CLI: ln(Step length) |  | - | - | - | - |
|  |  | LIS: ln(Step length) |  | - | - | - | - |
|  |  | PT: ln(Step length) |  | - | - | - | - |
|  |  | Poly: ln(Step length) |  | - | - | - | - |
|  |  | RIP:ln(Step length) |  | 0 | 0 | 4 | 4 |
| Moose | Selection | Conifer |  | 4 | 5 | 9 | 18 |
|  |  | Deciduous/Mixedwood |  | 4 | 3 | 10 | 17 |
|  |  | Wetland |  | 4 | 7 | 7 | 18 |
|  |  | ln(Distance to LF) |  | 0 | 5 | 13 | 18 |
|  |  | ln(Distance to Poly) |  | 1 | 3 | 14 | 18 |
|  |  | ln (Distance to RIP) |  | 6 | 2 | 10 | 18 |
|  | Movement | CLI: ln(Step length) |  | 0 | 3 | 0 | 3 |
|  |  | LIS: ln(Step length) |  | 0 | 1 | 1 | 2 |
|  |  | PT: ln(Step length) |  | - | - | - | - |
|  |  | Poly: ln(Step length) |  | 0 | 1 | 1 | 2 |
|  |  | RIP:ln(Step length) |  | 2 | 8 | 8 | 18 |

**Table S7.2**: Individual caribou responses to human disturbances and natural habitat and 95% Confidence Intervals. CLI = Conventional seismic, low-grade roads and ice-roads, LIS = low-impact seismic, PT = pipelines and transmission lines, Poly = polygonal disturbances and RIP = riparian habitat.

| **Variable** | **C004** | |  | **C005** | |  | **C007** | |  | **C009** | |
| --- | --- | --- | --- | --- | --- | --- | --- | --- | --- | --- | --- |
|  | **Beta** | **CI** |  | **Beta** | **CI** |  | **Beta** | **CI** |  | **Beta** | **CI** |
| Conifer | 0.507 | 0.453 |  | -0.369 | 0.363 |  | 0.198 | 0.174 |  | 0.001 | 2.105 |
| Deciduous/Mixedwood | 0.751 | 1.370 |  | -9.549 | 2058.634 |  | -0.376 | 0.287 |  | -0.193 | 2.823 |
| Wetland | 0.707 | 0.424 |  | -0.184 | 0.288 |  | - | - |  | 0.156 | 2.094 |
| ln(Distance to LF) | 0.087 | 0.156 |  | 1.151 | 0.765 |  | 0.875 | 3.043 |  | -0.193 | 0.230 |
| ln(Distance to Poly) | -0.052 | 1.703 |  | 1.703 | 4.523 |  | 0.035 | 6.928 |  | 21.642 | 12.726 |
| ln (Distance to RIP) | 0.626 | 0.316 |  | 0.564 | 0.718 |  | -0.116 | 0.272 |  | 0.792 | 0.519 |
| ln(Step length) | -0.621 | 0.041 |  | -0.714 | 0.042 |  | -0.514 | 0.040 |  | -0.697 | 0.041 |
| Cos (Turnangle) | -1.332 | 0.188 |  | -1.205 | 0.188 |  | -1.124 | 0.197 |  | -1.241 | 0.176 |
| LogSLCosTA | 0.055 | 0.028 |  | 0.318 | 0.058 |  | 0.223 | 0.055 |  | 0.353 | 0.055 |
| CLI: ln(Step length) | - | - |  | - | - |  | - | - |  | - | - |
| LIS: ln(Step length) | 0.130 | - |  | - | - |  | - | - |  | - | - |
| PT: ln(Step length) | - | - |  | - | - |  | - | - |  | - | - |
| Poly: ln(Step length) | - | - |  | - | - |  | - | - |  | - | - |
| RIP:ln(Step length) | 0.124 | 0.065 |  | 0.073 | 0.464 |  | -0.032 | 0.153 |  | 0.216 | 0.267 |

**Table S7.3**: Predator individual responses to human disturbances and natural habitat. Negative and positive are defined as model coefficients with 95% CIs that do not overlap zero, and neutral as model coefficients with 95% CIs that overlap zero. CLI = Conventional seismic, low-grade roads and ice-roads, LIS = low-impact seismic, PT = pipelines and transmission lines, Poly = polygonal disturbances and RIP = riparian habitat.

| **Species** | **Component** | **Variable** | **Response** | | | |
| --- | --- | --- | --- | --- | --- | --- |
|  |  |  | **-** | **+** | **=** | **n** |
| Bear | Selection | Conifer | 9 | 6 | 19 | 34 |
|  |  | Deciduous/Mixedwood | 2 | 15 | 17 | 34 |
|  |  | Wetland | 12 | 5 | 17 | 34 |
|  |  | ln(Distance to LF) | 8 | 1 | 25 | 34 |
|  |  | ln(Distance to Poly) | 7 | 2 | 25 | 34 |
|  |  | ln (Distance to RIP) | 12 | 4 | 18 | 34 |
|  | Movement | CLI: ln(Step length) | 0 | 12 | 1 | 13 |
|  |  | LIS: ln(Step length) | 0 | 0 | 2 | 2 |
|  |  | PT: ln(Step length) | 0 | 14 | 1 | 15 |
|  |  | Poly: ln(Step length) | 0 | 5 | 12 | 17 |
|  |  | RIP: ln(Step length) | 2 | 7 | 25 | 34 |
| Wolf | Selection | Conifer | 2 | 21 | 9 | 32 |
|  |  | Deciduous/Mixedwood | 4 | 16 | 12 | 32 |
|  |  | Wetland | 4 | 10 | 18 | 32 |
|  |  | ln(Distance to LF) | 14 | 3 | 15 | 32 |
|  |  | ln(Distance to Poly) | 9 | 3 | 20 | 32 |
|  |  | ln(Distance to Water) | 14 | 6 | 12 | 32 |
|  | Movement | CLI: ln(Step length) | 0 | 19 | 2 | 21 |
|  |  | LIS: ln(Step length) | 0 | 2 | 1 | 3 |
|  |  | PT: ln(Step length) | 0 | 14 | 0 | 14 |
|  |  | Poly: ln(Step length) | 5 | 3 | 14 | 22 |
|  |  | RIP: ln(Step length) | 0 | 26 | 6 | 32 |

**References:**

Avgar, T., Lele, S. R., Keim, J. L., & Boyce, M. S. (2017). Relative Selection Strength: Quantifying effect size in habitat- and step-selection inference. *Ecology and Evolution*, *7*(14), 5322–5330. doi:10.1002/ece3.3122

Demars, C. A., Auger-Methe, M., Schlagel, U., & Boutin, S. (2013). Inferring parturition and neonate survival from movement patterns of female ungulates: a case study using woodland caribou th e. *Ecology and Evolution*, *3*(12), 4149–4160. doi:10.1002/ece3.785

DeMars, C. A., & Boutin, S. (2017). Nowhere to hide: effects of linear features on predator-prey dynamics in a large mammal system. *Journal of Animal Ecology*, (87), 274–284. doi:10.1111/1365-2656.12760

Frair, J. L., Merrill, E. H., Visscher, D. R., Fortin, D., Beyer, H. L., & Morales, J. M. (2005). Scales of movement by elk (*Cervus elaphus*) in response to heterogeneity in forage resources and predation risk. *Landscape Ecology*, *20*(3), 273–287. doi:10.1007/s10980-005-2075-8

James, A., & Stuart-Smith, A. (2000). Distribution of caribou and wolves in relation to linear corridors. *The Journal of Wildlife Management*, *64*(1), 154–159.

Johnson, D. H. (1980). The comparison of usage and availability measurements for evaluating resource preference. *Ecology*, *61*(1), 65–71. doi:10.2307/1937156

Lindzey, F., & Meslow, E. (1977). Home range and habitat ese by black bears in southwestern Washington. *The Journal of Wildlife Management*, *41*(3), 413–425.

Muhly, T., Semeniuk, C., Massolo, A., Hickman, L., & Musiani, M. (2011). Human activity helps prey win the predator-prey space race. *PLoS One*, *6*(3), e17050.

Mumma, M. A., Gillingham, M. P., Johnson, C. J., & Parker, K. L. (2017). Understanding predation risk and individual variation in risk avoidance for threatened boreal caribou. *Ecology and Evolution*, *7*, 10266–10277. doi:10.1002/ece3.3563

Mysterud, A., & Ims, R. (1998). Functional responses in habitat use: Availability influences relative use in trade-off situations. *Ecology*, *79*(4), 1435–1441.

Newton, E. J., Patterson, B. R., Anderson, M. L., Rodgers, A. R., Vander Vennen, L. M., & Fryxell, J. M. (2017). Compensatory selection for roads over natural linear features by wolves in northern Ontario: Implications for caribou conservation. *PLoS ONE*, *12*(11), 1–21. doi:10.1371/journal.pone.0186525

Northrup, J. M., Pitt, J., Muhly, T. B., Stenhouse, G. B., Musiani, M., & Boyce, M. S. (2012). Vehicle traffic shapes grizzly bear behaviour on a multiple-use landscape. *Journal of Applied Ecology*, *49*, 1159–1167. doi:10.1111/j.1365-2664.2012.02180.x

Ofstad, E., Herfinal, I., Solberg, E., & Saether, B. (2016). Home ranges, habitat and body mass: simple correlates of home range size in ungulates. *Proceedings of the Royal Society B*, *283*, 1–8. doi:http://dx.doi.org/10.1098/rspb.2016.1234

Prokopenko, C. M., Boyce, M. S., & Avgar, T. (2016). Characterizing wildlife behavioural responses to roads using integrated step selection analysis. *Journal of Applied Ecology*, *54*, 470–479.

Severud, W., DelGiudice, G., Obermoller, T., Enright, T., R., W., & Forester, J. (2015). Using GPS collars to determine parturition and cause-specific mortality of moose calves. *Wildlife Society Bulletin*, *39*(3), 616–625. doi:10.1002/wsb.558

Theuerkauf, J., Jedrzejewski, W., Schmidt, K., & Gula, R. (2003). Spatiotemporal segregation of wolves from humans in the Bialowieza Forest (Poland). *The Journal of Wildlife Management*, *67*(4), 706–716.

Whittington, J., St. Clair, C. C., & Mercer, G. (2005). Spatial responses of wolves to roads and trails in mountain valleys. *Ecological Applications*, *15*(2), 543–553.

Zimmermann, B., Nelson, L., Wabakken, P., Sand, H., & Liberg, O. (2014). Behavioral responses of wolves to roads: scale-dependent ambivalence. *Behavioral Ecology*, *25*, 1353–1364. doi:10.1093/beheco/aru134
